# Optimization of Clinical Trial Design and Decision-Making for Heart Failure with Preserved Ejection Fraction (HFpEF): A Meta-Analysis Based on a Placebo Response Model

**DOI:** 10.1155/cdr/7087720

**Published:** 2025-10-06

**Authors:** Xiaoya Chen, Juan Yang, Ling Xu, Fang Yin, Jihan Huang, Yinghua Lv, Qingshan Zheng, Lujin Li

**Affiliations:** ^1^Center for Drug Clinical Research, Shanghai University of Traditional Chinese Medicine, Shanghai, China; ^2^State Key Laboratory of Integration and Innovation of Classic Formula and Modern Chinese Medicine (Shanghai University of Traditional Chinese Medicine), Shanghai, China

**Keywords:** clinical trial protocol optimization, HFpEF, MBMA, sample size estimation

## Abstract

**Objective:**

The aim of this study was to establish a placebo response model for efficacy indicators in heart failure with preserved ejection fraction (HFpEF) clinical trials, facilitating sample size estimation and trial optimization.

**Methods:**

PubMed, EMBASE, and Cochrane Library databases were searched systematically for placebo-controlled trials of HFpEF up to May 26, 2024. Using model-based meta-analysis (MBMA), we analyzed cardiovascular death or heart failure hospitalization, cardiovascular death, heart failure hospitalization, all causes of death, and changes from baseline in the 6-min walk distance (6MWD) of the placebo group. The final model simulated the placebo effect distribution for these indicators under varying scenarios.

**Results:**

A total of 29 studies and 14,302 participants were analyzed. A log-normal risk function was developed to describe four event rate indicators, and typical event rates for the placebo group over 6 years were simulated. After 1 year, rates for cardiovascular death or heart failure hospitalization (composite event), heart failure hospitalization, all causes of death, and cardiovascular death were 11.1%, 10.5%, 2.91%, and 2.33%, respectively. We used a linear model to describe the time–effect relationship of the change value of change from baseline in 6MWD, revealing typical changes at 1, 3, and 6 months as 1.47, 4.76, and 9.57 m, respectively. These values indicated that using the composite event as criteria could reduce the sample size by 500–12,000 cases, while 548 cases were sufficient for changes in 6MWD. The placebo effect model was also used as an external control in evaluating Sacubitril/Valsartan and exercise training efficacy.

**Conclusion:**

This placebo response model provides essential support for sample size estimation, trial optimization, and drug efficacy evaluation in future HFpEF clinical trials.

**Summary**

  This study systematically reviewed the efficacy endpoints in heart failure (HF) with preserved ejection fraction (HFpEF) clinical trials and established a pharmacodynamic model that describes the typical value distributions of four event occurrence rates in the placebo group and change from baseline in 6-min walk distance (6MWD) at different time points, which not only provided important references for sample size estimation to optimize clinical trial designs but also served as an external control standard, offering preliminary decision-making support for exploratory clinical studies that lack placebo controls.

**• Key findings**

∘ We established a log-normal risk function to describe four event rate indicators and simulated the typical event rates for the placebo group over 6 years and found that among the event incidence indicators, cardiovascular death or hospitalization for HF has a higher incidence and smaller variation, and choosing this indicator can reduce the sample size required for the trial compared to other events. Additionally, we used a linear model to describe the time effect characteristics of the change in 6MWD from baseline, with typical changes for placebo interventions at 1, 3, and 6 months being 1.47, 4.76, and 9.57 m, respectively.
∘ Sacubitril/valsartan did not significantly reduce the risk of cardiovascular death compared with placebo, and exercise training significantly increased 6MWD (26.58 m) compared with placebo, approaching clinically significant values.

## 1. Introduction

HF is a clinical syndrome caused by impaired cardiac function that fails to meet the body's metabolic demands. It is a leading contributor to global morbidity and mortality [1, 2]. Ejection fraction (EF) refers to the percentage of blood expelled from the ventricles during each heartbeat relative to the volume at end-diastole, most commonly measured in the left ventricle. Normal values typically range from 50% to 70%. HFpEF is a subtype of HF characterized by normal left ventricular ejection fraction (LVEF ≥ 50%) alongside inadequate diastolic filling [3, 4]. Its pathophysiology is complex and multifactorial, often occurring with comorbidities such as obesity, diabetes, and hyperlipidemia, and remains incompletely understood [5, 6]. The prevalence of HFpEF continues to rise, particularly among the elderly, now affecting up to 32 million individuals worldwide. Patients with HFpEF experience a poor prognosis, with high rates of hospitalization and rehospitalization—averaging approximately 1.4 hospital admissions per year and an annual mortality rate of 15% [7]. The economic burden from acute care and chronic management is substantial [8, 9]. According to the “2023 ACC Expert Consensus Decision Pathway on Management of Heart Failure With Preserved Ejection Fraction,” there is currently no universally accepted standard therapy for HFpEF [10]. Addressing this therapeutic gap is an urgent priority, underscoring the need for the development of more effective and safer pharmacologic interventions.

Published clinical trials for HFpEF commonly incorporate endpoints assuming cardiopulmonary function and event occurrence rates. The 6-min walk test (6MWT), a method for evaluating cardiopulmonary capacity, is widely applied due to its simplicity, high tolerability, and ability to reflect patients' functional status in daily life. Additionally, it serves as a prognostic indicator for hospitalization and mortality risk in HF patients [11, 12]. However, the 6MWT is subject to considerable measurement variability influenced by differences in body size, gender, muscle mass, and inconsistencies in test administration [13]. Event-based endpoints typically monitored include cardiovascular death or hospitalization for HF, cardiovascular death, all causes of death, and hospitalization for HF [14–19]. In 2021, the FDA approved empagliflozin and dapagliflozin for the treatment of adult patients with HFpEF, with supporting clinical trials utilizing event occurrence rates as primary endpoints [14, 15]. However, the low incidence of these events in early-stage HFpEF necessitates large sample sizes to achieve statistical power, thereby increasing trial complexity and cost. As a result, some pharmaceutical companies have discontinued drug development efforts for HFpEF, impeding innovation and limiting therapeutic advances for this critically underserved patient population.

Clinical trials for HFpEF often employ placebo controls, necessitating accurate quantification of the placebo effect to inform sample size estimation across various study scenarios. This enables scientifically grounded selection of outcome measures and trial configurations. Additionally, constructing historical placebo external control groups provides essential benchmarks for potential drug screening in single-arm studies and real-world research. Model-based meta-analysis (MBMA) can precisely quantify the time–effect relationship of the placebo response and identify various factors influencing clinical trials, which helps explore the sources of heterogeneity between trials [20]. In this study, MBMA was applied to quantitatively analyze the incidence rates of key clinical events within placebo arms and the change from baseline in 6MWD across HFpEF trials. These quantitative results will be used for sample size estimation and to provide external controls for single-arm trials, thereby offering effective strategies and methods for the clinical development of HFpEF drugs.

## 2. Methods

This study followed the Preferred Reporting Items for Systematic Reviews and Meta-analyses and was registered in PROSPERO (CRD42024616421). The data for this study were obtained from literature reports.

### 2.1. Study Selection

A systematic literature search was conducted in three databases: PubMed, EMBASE, and the Cochrane Library, to collect information on placebo-controlled randomized trials concerning the pharmacological treatment of HFpEF. The search covered the period from the inception of each database to May 26, 2024, and was restricted to articles published in English. A detailed search strategy is provided in Table S1. To minimize the risk of omitting relevant studies, we also manually searched the references cited in related systematic reviews.

### 2.2. Inclusion and Exclusion Criteria

The inclusion criteria were randomized, double-blind, placebo-controlled trials in patients with HFpEF in which at least one of four event rate measures or changes in 6MWD was reported. The four event rate measures include HF hospitalization (hospitalization due to HF), cardiovascular death (deaths directly caused by heart or vascular diseases, including acute myocardial infarction, sudden cardiac death, HF, stroke, fatal arrhythmias, deaths related to cardiovascular surgery or interventional therapy, and so on), all causes of death (death from any cause, both vascular and nonvascular causes), and cardiovascular death or HF hospitalization (either cardiovascular death or HF hospitalization occurs) [21, 22]. Changes in 6MWD means change from baseline in 6MWD. Detailed inclusion and exclusion criteria are provided in Methods S1.

### 2.3. Data Extraction

Information such as literature characteristics, trial design characteristics, participants' baseline characteristics, and clinical outcomes (event rates and 6MWD at each follow-up point) was extracted. The detailed data extraction method is described in Methods S1.

If efficacy data in the literature were presented in graphical form, they were digitized using Engauge Digitizer software, and the average of the values extracted by the two researchers was used as the final data for analysis. If the discrepancy in data extraction between the two researchers exceeded 2%, the data extraction was repeated.

### 2.4. Literature Quality Assessment

The quality of each study was independently assessed using the Cochrane risk-of-bias Tool 2 by two authors (X.C. and J.Y.) independently. Any differences were resolved through discussion with another author (L.L.). The detailed evaluation results are described in Table S4 and Figure S2.

### 2.5. Statistical Analysis

#### 2.5.1. Model Building

This study aims to develop a time–effect model for the placebo group, focusing on five endpoints: [1] cardiovascular death or hospitalization for HF, [2] cardiovascular death, [3] hospitalization for HF, [4] all causes of death, and [5] change from baseline in 6MWD. Model development includes structural, random effects, and covariate models. Event-based endpoints will be characterized using survival analysis, enabling time-to-event modeling of incidence rates, while a linear model will be applied to quantify the expected temporal increase in 6MWD. Further details are provided in Methods S2.

#### 2.5.2. Model Assessment

Model diagnostic plots, visual predictive check (VPC), and sampling importance resampling (SIR) methods were used to evaluate goodness of fit, model estimation ability, and stability. Details are provided in Methods S3.

#### 2.5.3. Simulation of Typical Efficacy in the Placebo Group

Based on the established models for various placebo response metrics, we can simulate the distribution of typical efficacy values for the placebo group at different time points. These metrics refer to the five aforementioned clinical endpoints. If a covariate model has been developed, we will also simulate the distribution of efficacy values for the placebo group across different levels of covariates for each efficacy indicator.

#### 2.5.4. Model Application: Sample Size Estimation

The model established in this study can provide accurate information for the sample size estimation of HFpEF clinical trials. This study will estimate the sample size corresponding to each pharmacodynamic index according to different simulation scenarios. For the estimation of sample sizes related to event occurrence rates, the two-sided logrank test method was used to estimate the sample sizes required to achieve 80% or 90% power at a 0.05 significance level to detect a hazard ratio (HR) between 0.45 and 0.80, when the placebo group incident rate uses the model simulation results at 1 year, and the simulation scenarios include different accrual durations (AT = 12, 18, and 24 months) and varying total study durations (TT = 24, 30, 36, 42, 48, 54, and 60 months). For the change from baseline in 6MWD, the two-sided *T*- test method was used to estimate the sample sizes required to achieve 80% power at a 0.05 significance level to detect differences between groups of 30, 50, or 70 m, when the standard deviation (SD) of efficacy is 100, 125, or 150 m.

Both of them, the drug group and the placebo group were randomly assigned at 1:1.

#### 2.5.5. Model Application: Providing External Controls for Clinical Trials

The PARAGON-HF trial, a randomized, double-blind, positive control trial conducted among patients with HFpEF [23], aimed to evaluate the efficacy of sacubitril/valsartan for the treatment of HFpEF. Due to the fact that most patients had received treatment with renin–angiotensin system inhibitors prior to enrollment, a placebo-controlled trial design was not feasible. Valsartan, a representative of renin–angiotensin system inhibitors, was therefore selected as the control drug. The study concluded that sacubitril/valsartan did not demonstrate significant benefits compared to valsartan alone. A meta-analysis showed that sacubitril/valsartan had an advantage over valsartan in reducing the combined outcome risk of HF decompensation and all causes of death in patients with HFpEF [24]. Our study will use historical placebo data as an external control to compare the efficacy of sacubitril/valsartan against historical placebo groups, in order to further assess the relative efficacy advantage of sacubitril/valsartan.

Exercise intolerance is a major symptom of HFpEF, making it crucial to improve exercise capacity in these patients. However, there has been no quantitative analysis of the efficacy of exercise training on the exercise capacity of HFpEF patients. Borlaug et al. conducted a multicenter, randomized, double-blind controlled trial that included HFpEF patients who underwent exercise training while receiving either 40 mg of inorganic nitrite or a placebo [25]. The primary pharmacodynamic endpoint was the change from baseline in 6MWD. By comparing the results of the exercise training plus placebo group from this study with the historical placebo group effects established in our research, we can preliminarily assess the benefit of exercise training relative to placebo.

#### 2.5.6. Software

Model construction was performed using NONMEM 7.4 (ICON Development Solutions, United States), with parameter estimation conducted via the first-order conditional estimation with interaction method (FOCE-I). Model simulations and plotting were carried out using R software (Version 4.0.3, the R Foundation for Statistical Computing, Vienna, Austria). Sample size estimation was conducted using PASS2022 software (Power Analysis and Sample Size Software 2022).

## 3. Results

### 3.1. Characteristics of the Included Studies

**Figure 1:**
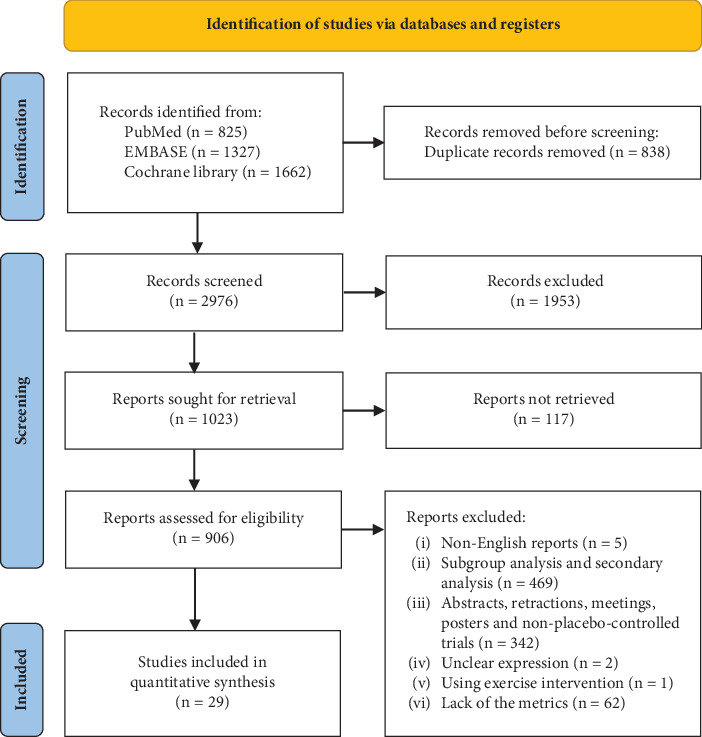
Flow chart demonstrating the inclusion and exclusion of studies into the analysis.

A total of 3814 studies were initially retrieved. Following screening, 29 studies met the inclusion criteria and were incorporated into the analysis, involving 14,302 participants. Among these, four studies (13.8%) reported on cardiovascular death or hospitalization for HF, involving 9355 participants; five studies (17.2%) reported on cardiovascular death, with 10,333 participants; four studies (13.8%) reported on all causes of death, covering 9907 participants; five studies (17.2%) reported on hospitalization for HF, involving 8310 participants; and 24 studies (82.8%) reported change from baseline in 6MWD, with a total of 2974 participants. The flowchart of the literature selection process is shown in Figure 1. A comprehensive list of included studies, along with baseline characteristics and results from the quality assessment, is provided in Tables S2 and S3 and Figure S1.

### 3.2. Model Establishment and Assessment

Based on the minimization of the objective function value (OFV) and the precision of model parameter estimates, the log-normal hazard function was ultimately selected to describe the incidence rates of four clinical events. A linear model appropriately captured the time course of change from baseline in 6MWD. Covariate screening revealed no significant effects of age, body mass index (BMI), sex (proportion of female participants), or baseline LVEF on model parameters; therefore, the base model was retained as the final model.

**Figure 2:**
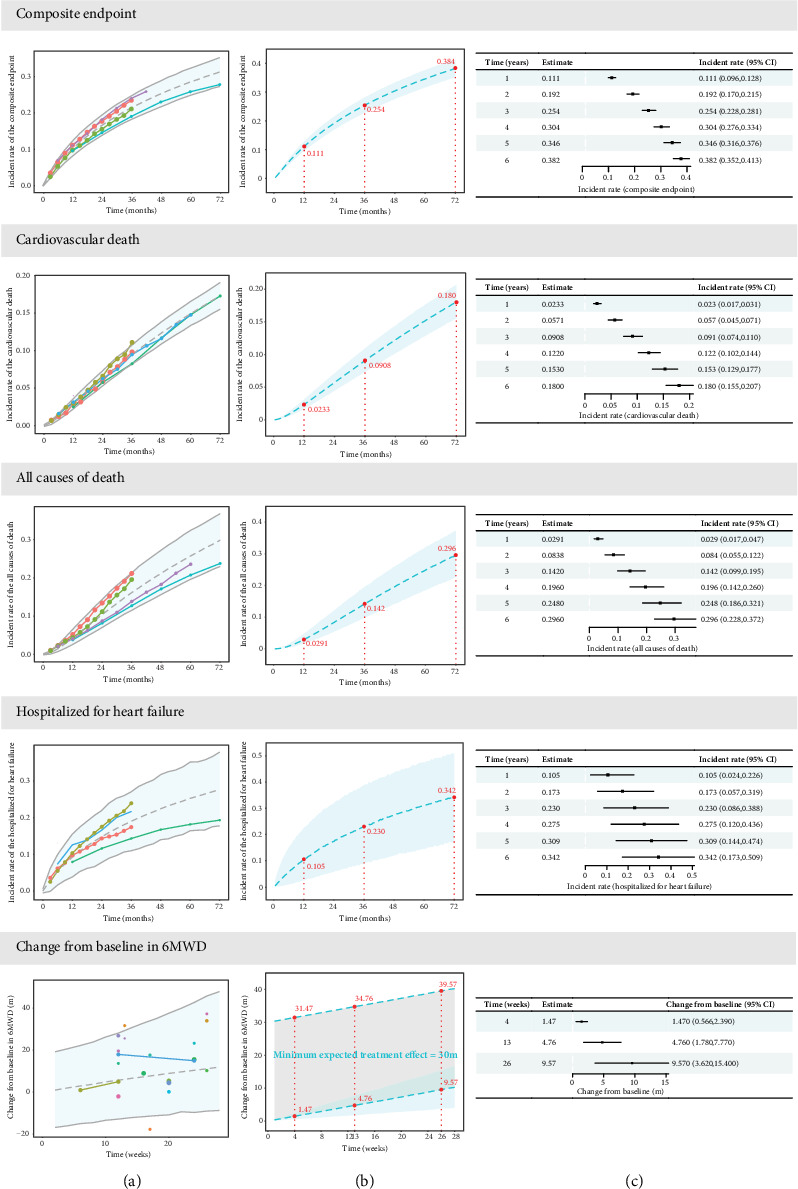
The visual predictive check plots and model prediction of typical values for the placebo effect. Composite endpoint: Cardiovascular death or hospitalization for heart failure. (a) Visual predictive check plots of the final model. The dots in the figure represent measurements, and the size of each dot is proportional to the sample size in each trial. The shaded areas represent the 90% CI predicted by the model, and the dashed lines represent the median. As can be seen from the figure, most of the measuring points fall within the 90% CI range predicted by the model. This shows that the model has good forecasting ability. (b) Simulation of typical values of the placebo effect. The dashed blue line is the typical value predicted by the placebo model, and the shadow is the 95% CI of the typical value. For the change from baseline in 6MWD, the gray shadow represents the clinical minimum effective value of 30 m, and the blue thick dashed line represents the clinical minimum effective value. (c) Typical values and 95% CI of the placebo effect at different time points.

Estimates of the model parameters are summarized in Table S5. The parameter medians obtained via SIR closely matched those estimated from the original dataset, indicating robust estimation. Goodness-of-fit diagnostics (Figure S3) showed that the model fits the observed data well, with no significant bias. The VPC plots reveal that the majority of the observed data fall within the 90% CIs predicted by the model, suggesting that the model possesses good predictive ability (Figure 2a).

### 3.3. Distribution of Typical Event Incidence Rates in the Placebo Group

Based on the model developed, this study simulated the distribution of typical values for the incidence rates of four types of events over a 6-year period in historical placebo groups (Figure 2b,c). The incidence rates of cardiovascular death or hospitalization for HF at 1, 3, and 6 years were 11.1%, 25.4%, and 38.2%, respectively. The annual incidence rates of these events decreased over time, with Year 3 and Year 6 rates being 6.2% and 3.6%, respectively.

The incidence rates of hospitalized for HF were 10.5% at 1 year, 23.0% at 3 years, and 34.2% at 6 years. As time progressed, the annual incidence rates for cardiovascular death or HF hospitalization gradually decreased, with Year 3 and Year 6 rates at 5.7% and 3.3%, respectively.

The incidence rates for all causes of death were 2.91% at 1 year, 14.2% at 3 years, and 29.6% at 6 years. The annual incidence rate for all causes of death was lower in the first year, stabilizing at approximately 5% thereafter.

The incidence rates of cardiovascular death were 2.33% at 1 year, 9.08% at 3 years, and 18.0% at 6 years, with an approximate annual rate of 3%.

At matched time points, the incidence rates of events from highest to lowest were as follows: cardiovascular death or hospitalized for HF, hospitalized for HF, all causes of death, and cardiovascular death. For example, at the 3-year mark, the incidence rates for cardiovascular death or hospitalized for HF were, respectively, 2.4%, 11.2%, and 16.3% higher than those for hospitalization for HF, all causes of death, and cardiovascular death.

In terms of precision of incidence rate estimation, at the 3-year mark, the 95% confidence intervals (CIs) for the typical values of the incidence rates for cardiovascular death or hospitalized for HF, hospitalized for HF, all causes of death, and cardiovascular death were, respectively, 89.8%–110.6%, 37.6%–168.7%, 69.5%–137.3%, and 81.6%–121.1% relative to the median value. The highest estimation precision was observed for the incidence rates of cardiovascular death or hospitalized for HF.

### 3.4. Distribution of Typical Values for 6MWD in the Placebo Group

Based on the final model, the distribution of typical changes from baseline in 6MWD at specified time points in the placebo group was simulated (Figure 2b,c). The typical change from baseline in 6MWD at 1, 3, and 6 months was 1.47 m (95% CI: 0.566, 2.39), 4.76 m (95% CI: 1.78, 7.77), and 9.57 m (95% CI: 3.62, 15.4), respectively.

### 3.5. Sample Size Estimation of Different Pharmacodynamic Indexes Under Different Conditions

**Figure 3:**
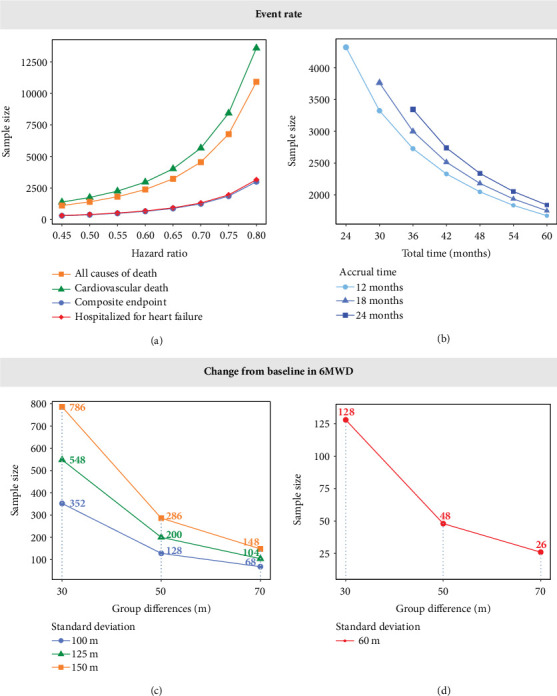
Sample size estimation results under different conditions. (a, c) The sample size estimation results of each efficacy index under normal conditions. (b, d) The recommended sample size estimation results ((a) power = 0.8, alpha = 0.05, AT = 18 months, TT = 36 months; (b) power = 0.8, alpha = 0.05, HR = 0.8; (c) power = 0.8, alpha = 0.05; and (d) power = 0.8, alpha = 0.05).

For event rate endpoints, assuming a power of 0.80, a two-sided alpha of 0.05, AT of 18 months, and TT of 36 months, required sample sizes were calculated across HRs of 0.6, 0.7, and 0.8. Incidence rates at 1 year for the four outcomes were set to typical values: composite outcome (0.111), cardiovascular death (0.0233), all causes of death (0.0291), and hospitalization for HF (0.105). The estimated sample sizes for the composite endpoint of cardiovascular death or hospitalization for HF were 647, 1243, and 2999 for HRs of 0.6, 0.7, and 0.8, respectively. For hospitalization for HF alone, the required sample sizes were slightly higher: 682, 1310, and 3159. Sample size requirements were markedly greater for single endpoints such as all cause of death (2381, 4550, and 10,922 cases) and cardiovascular death (2966, 5666, and 13,597 cases). Taking HR = 0.8 as an example, the sample size for cardiovascular death or hospitalization for HF was reduced by 160, 7923, and 10,598 cases compared to the requirements for hospitalization for HF, all causes of death, and cardiovascular death, respectively (Figure 3a), highlighting the efficiency gains of using composite outcomes in endpoint selection. The study showed that, with fixed conditions (power = 0.8, HR = 0.8, and TT = 36 months), shortening AT significantly reduced the sample size. Taking the rate of cardiovascular death or hospitalization for HF as an example, shortening AT from 18 to 12 months resulted in a reduction of 272 participants. Similarly, when other conditions remain unchanged (power = 0.8, HR = 0.8, and AT = 18), extending TT can also significantly reduce the sample size. Similarly, taking the rate of cardiovascular death or hospitalization due to HF as an example, when TT is increased from 36 to 48 months, the sample size can be reduced by 819 cases (Figure 3b).

For the change from baseline in 6MWD, sample size calculations were conducted using a power of 0.80, a SD of 125 m (as reported in the literature), and a between-group difference of 30 m (the minimum clinically meaningful difference) [26]. Under these assumptions, the estimated sample size required was approximately 548 participants (Figure 3c). With all other parameters held constant, reducing the SD from 125 to 60 m (the lowest value reported in the included literature) would lower the required sample size by 420 participants (Figure 3d).

### 3.6. Historical External Controls Were Provided for the Placebo Group

**Figure 4:**
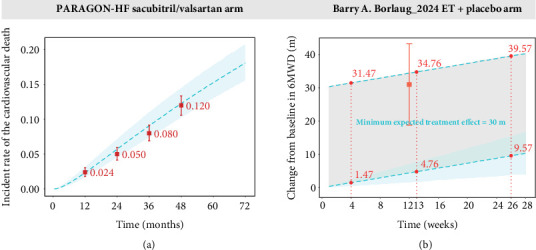
(a, b) Assess the efficacy of the test group using the typical distribution of the placebo effect as an external control. ET: exercise training. The square points in the figure represent the measured efficacy values of the test group, while the error bars indicate the 95% CIs of these measurements. The blue shaded area represents the 95% CIs of the model-predicted typical values of the placebo effect, with the blue dashed line within the shaded area indicating the typical placebo value. In (a), the thick blue dashed line represents the clinically minimal effective value, and the gray shaded area indicates the interval below the clinically minimal effective value.

The 1-, 2-, and 3-year rates of cardiovascular death in the sacubitril/valsartan arm in the PARAGON-HF study were 2.43%, 5.05%, and 8.04%, respectively. In comparison, the corresponding rates in the historical placebo cohort were 2.33%, 5.71%, and 9.08% (Figure 4a). These data indicate that cardiovascular mortality rates were closely aligned between the sacubitril/valsartan and historical placebo groups, suggesting no substantial survival benefit associated with sacubitril/valsartan over the observed timeframe.

In the study conducted by Borlaug et al., the change from baseline in 6MWD at 12 weeks in the exercise training group was 31 m, compared with 4.42 m in the historical placebo group. This represents a 26.58-m greater improvement in the exercise group relative to the placebo (Figure 4b), highlighting the potential efficacy of exercise training in enhancing functional capacity among patients with HFpEF.

## 4. Discussion

This study summarized published placebo-controlled clinical trials for HFpEF and conducted modeling analyses on the primary efficacy endpoints of the placebo groups. This analysis quantitatively characterized the temporal changes and distribution ranges of various efficacy indicators, providing valuable information for subsequent HFpEF clinical trials. The event occurrence rate is a key hard endpoint in HFpEF clinical trials. At present, the event incidence indicators included in clinical trials are as follows: cardiovascular death or hospitalization for HF, cardiovascular death, all causes of death, and hospitalization for HF rates. Among these, the composite endpoint of cardiovascular death or hospitalization for HF has a higher incident rate than the other three individual events. For example, at 3 years, the occurrence rate of cardiovascular death or hospitalization for HF is 25.4%, which is 2.4%, 11.2%, and 16.3% higher than those of cardiovascular death, all causes of death, and hospitalization for HF, respectively. It is evident that the majority of occurrences of cardiovascular death or hospitalization for HF are related to hospitalization for HF. Furthermore, in terms of numerical precision, the variability of the cardiovascular death or hospitalization for HF events across trials is the lowest, with the estimated typical value of the placebo group having a 95% CI of 89.8%–110.6% relative to the median value. This suggests good precision and stability, making it more suitable as an efficacy evaluation indicator compared to the other three endpoints.

When the event occurrence rate is higher, the sample size required to achieve statistical differences between the two groups in clinical trials becomes smaller. However, due to the overall low event occurrence rate in HFpEF clinical trials, the estimated sample size tends to be larger. Currently, completed HFpEF clinical trials that use event occurrence rates as the primary efficacy endpoint typically have sample sizes ranging from 2000 to 3000 subjects (Table S6), which increases the difficulty and cost of implementing these trials, leading many pharmaceutical companies to terminate drug development in the HFpEF treatment area. This study provided the distribution of four event occurrence rates in the placebo group at different time points during HFpEF clinical trials, allowing for the estimation of sample sizes under various trial conditions, thus offering a reference for optimizing clinical trial designs.

In several large clinical studies, the HRs for event occurrence rates of investigational drugs compared to placebo range from 0.79 to 0.89 (with a median of 0.86). The enrollment period varies from 16 to 65.6 months (median 35 months), and the follow-up duration ranges from 26.2 to 49.5 months (median 32.1 months) [14–19]. Assuming that the endpoint indicator is the occurrence rate of cardiovascular death or hospitalization for HF, the standard conditions for sample size estimation are set as follows: power = 0.8, HR = 0.8, AT = 24 months, and TT = 36 months. When AT is reduced from 24 to 12 months, or TT is extended from 36 to 48 months, the required sample sizes can be reduced by 617 and 1006, respectively. Of course, shortening AT implies that more trial centers are needed to maintain the same number of participants, while extending TT increases the risk of participant dropout and overall trial costs. Although these measures can reduce the sample size, they also complicate the organization and implementation of the trial, requiring researchers to weigh these factors carefully. A reduction in HR for the trial group compared to the placebo group is a significant factor that can substantially decrease the sample size without increasing trial difficulty. Currently, the HR for HFpEF treatment drugs relative to placebo is approximately 0.8; if a new mechanism drug achieves an HR of 0.7 relative to placebo, the sample size could be reduced from 2999 to 1387 (Figure S4).

When estimating sample size, it is also necessary to know the annual occurrence rate of events in the placebo group. This study found that different event occurrence rates vary unevenly over time. Taking the occurrence rate of cardiovascular death or hospitalization for HF as an example, the model results of this study indicate that the annual occurrence rates of this composite endpoint at 1, 3, and 6 years are 11.1%, 6.2%, and 3.6%, respectively. Since the model developed in this study can predict event occurrence rates at different time points, it can provide more accurate and reliable information on the occurrence rates of events in the placebo group for sample size estimation.

It should be noted that in several large-scale trials included in the analysis, the DELIVER trial defined the complex event as HF worsening or cardiovascular death (Table S7). However, considering that the article defines “HF worsening” as “hospitalization due to HF or emergency visits due to HF,” we also incorporated the results of the DELIVER trial into the modeling dataset. Subsequently, the rationality of this inclusion was validated through the results of a sensitivity analysis (Table S8).

In the clinical trials included in this study, 82.8% reported changes from baseline in 6MWD, particularly in exploratory studies with small sample sizes. This study also conducted a quantitative analysis of the changes in 6MWD from baseline in the placebo group over time. The results indicated that the baseline change value of 6MWD in the placebo group increased linearly over time, increasing by approximately 4.41 m every 3 months. Given that the placebo group included background treatments, this increase is primarily attributed to the effects of such therapies.

Based on the constructed changes in 6MWD from baseline in the placebo group, this study calculated the sample size required for using the change in 6MWD from baseline as the primary efficacy endpoint under different scenarios. Assuming a power of 0.8 and a SD of 125 m (the weighted average of the SDs from included literature), and with the treatment group expected to improve 6MWD by approximately 30 m relative to the placebo group (the minimum clinically meaningful difference), the required sample size would be 548 participants. Among the included studies, the sample sizes ranged from 41 to 772 (median 126), with the majority being below this value. This is largely due to the fact that most studies were exploratory and did not conduct strict sample size estimations for changes in 6MWD from baseline.

Additionally, the interindividual variability in changes in 6MWD from baseline was considerable, with the relative standard deviation (RSD) of the trial endpoints in the included studies ranging from 2.5% to 273%. Previous studies have reported that such large interindividual variability in changes in 6MWD may be related to psychological factors, weight, age, sex, and measurement techniques. Standardizing measurement procedures and controlling for confounding variables can help reduce interindividual variability, thereby potentially decreasing the sample size [27]. For example, if the SD is reduced to 60 m (the minimum SD found in the literature), while other conditions remain constant, the required sample size could be reduced to 128.

Compared to event occurrence rates, using change from baseline in 6MWD as the primary efficacy endpoint can significantly reduce the required sample size. However, the acceptability of change from baseline in 6MWD as a surrogate indicator for event occurrence rates has not yet been recognized by regulatory authorities, and there have been no successful cases of drug approvals based on this approach. It is recommended that future research conduct a correlation analysis between the change from baseline in 6MWD and the rates of cardiovascular death or hospitalization for HF. This would provide scientific evidence for the use of change from baseline in 6MWD as a surrogate endpoint in HFpEF clinical trials. Such an approach could greatly reduce the complexity of HFpEF clinical trials and accelerate drug development in this field.

The model constructed in this study can be used not only for sample size estimation but also as an external control to provide a rough standard for assessing the effectiveness or degree of effectiveness of interventions in studies lacking placebo controls. By comparing the incidence of cardiovascular death events in the sacubitril/valsartan trial group with typical values from historical placebo groups, this study found that the incidence of cardiovascular death events in the sacubitril/valsartan group was nearly consistent with that of the historical placebo groups. Although interstudy variability may exist, this conclusion requires further validation through rigorous randomized controlled trials (RCTs). Nevertheless, the results suggest that sacubitril/valsartan may not offer a significant advantage in reducing cardiovascular death risk, even if there is a reduction effect, as a particularly large sample size may be necessary to achieve statistical significance.

Additionally, this study compared the change from baseline in 6MWD during exercise training with historical placebo groups and found that the exercise training group increased by 26.58 m at 12 weeks compared to historical placebo groups. This value is close to the threshold for clinical significance and warrants further validation through RCTs. The purpose of using external controls is not to replace clinical trials but to identify promising interventions with potential therapeutic value from multiple exploratory studies, which can then be rigorously evaluated in subsequent RCTs. This approach can improve the efficiency of trial selection and reduce the risk of false-positive results.

The study has several limitations. Firstly, the modeling analysis was conducted based on literature data at the summary level. The reported potential influencing factors were presented as average values, resulting in a narrow range of variability for these factors and no statistically significant associations being identified. Secondly, due to the limitations of the available literature, many potential influencing factors were not reported, limiting the scope of our analysis. Thirdly, this study included a relatively small number of studies that reported the event incidence rates, which may limit the generalizability of the constructed model and suggest that the results should be interpreted with caution. Finally, only English-language publications were included, which may introduce publication bias.

## 5. Conclusions

This study systematically reviewed the efficacy endpoints in HFpEF clinical trials and established a pharmacodynamic model characterizing the typical value distributions of four event occurrence rates in placebo groups, as well as changes from baseline in 6MWD across multiple time points. This model provides valuable references for sample size estimation to optimize clinical trial designs and serves as an external control standard, offering preliminary decision-making support for exploratory clinical studies without placebo controls.

## Data Availability

The data that supports the findings of this study are available in the supporting information of this article.

## Supporting Information

Supporting Information Additional supporting information can be found online in the Supporting Information section. Table S1: Search strategy. Table S2: Basic information of the included studies. Table S3: The list of included studies. Figure S1: The feature map of the included studies. Table S4: The results of the literature assessment. Figure S2: Risk of bias assessment chart. Table S5: Estimated parameters for the placebo response model. Figure S3: The goodness plots of the final model. Table S6: Basic information of HFpEF clinical trials with event incidence as the primary outcome measure. Table S7: The definition of composite outcome events included in the trials. Table S8: The results of the sensitivity analysis of the composite outcome indicators. Figure S4: Sample size estimation results under different conditions (taking cardiovascular death or hospitalized for heart failure as an example). Methods S1: inclusion and exclusion criteria and data extraction. Methods S2: Model building. Methods S3: Model assessment method. Methods S4: Model NONMEM codes.
