# Supplementary material for: Optimization of Clinical Trial Design and Decision-Making for Heart Failure with Preserved Ejection Fraction (HFpEF): A Meta-Analysis Based on a Placebo Response Model
Source: Cardiovasc Ther. 2025 Oct 6;2025:7087720. doi: 10.1155/cdr/7087720 (PMC12517979; doi:10.1155/cdr/7087720)
Supplement: Supporting Information — Additional supporting information can be found online in the Supporting Information section. Table S1: Search strategy. Table S2: Basic information of the included studies. Table S3: The list of included studies. Figure S1: The feature map of the included studies. Table S4: The results of the literature assessment. Figure S2: Risk of bias assessment chart. Table S5: Estimated parameters for the placebo response model. Figure S3: The goodness plots of the final model. Table S6: Basic information of HFpEF clinical trials with event incidence as the primary outcome measure. Table S7: The definition of composite outcome events included in the trials. Table S8: The results of the sensitivity analysis of the composite outcome indicators. Figure S4: Sample size estimation results under different conditions (taking cardiovascular death or hospitalized for heart failure as an example). Methods S1: inclusion and exclusion criteria and data extraction. Methods S2: Model building. Methods S3: Model assessment method. Methods S4: Model NONMEM codes. [file 7087720.f1.docx]

**Supplementary Materials**

Supplementary Table S1: Search strategy.

Supplementary Table S2: Basic information of the included studies.

Supplementary Table S3: The list of included studies.

Supplementary Figure S1: The feature map of the included studies.

Supplementary Table S4: The results of the literature assessment.

Supplementary Figure S2: Risk of bias assessment chart.

Supplementary Table S5: Estimated parameters for the placebo response model.

Supplementary Figure S3: The goodness plots of the final model.

Supplementary Table S6: Basic information of HFpEF clinical trials with event incidence as the primary outcome measure.

Supplementary Table S7: The definition of composite outcome events included in the trials.

Supplementary Table S8: The results of the sensitivity analysis of the composite outcome indicators.

Supplementary Figure S4: Sample size estimation results under different conditions (taking cardiovascular death or hospitalized for heart failure as an example).

Supplementary Methods S1: Inclusion and exclusion criteria and data extraction.

Supplementary Methods S2: Model building.

Supplementary Methods S3: Model assessment method.

Supplementary Methods S4: Model NONMEM codes.

**Supplementary Table S1: Search strategy.**

Pubmed

| **NO.** | **Query** | **Results** |
| --- | --- | --- |
| #1 | "HFpEF"[All Fields] | 5780 |
| #2 | ("heart failure"[MeSH Terms] OR ("heart"[All Fields] AND "failure"[All Fields]) OR "heart failure"[All Fields]) AND ("preservation, biological"[MeSH Terms] OR ("preservation"[All Fields] AND "biological"[All Fields]) OR "biological preservation"[All Fields] OR "preservation"[All Fields] OR "preserved"[All Fields] OR "preservations"[All Fields] OR "preserve"[All Fields] OR "preserves"[All Fields] OR "preserving"[All Fields]) AND ("eject"[All Fields] OR "ejected"[All Fields] OR "ejecting"[All Fields] OR "ejection"[All Fields] OR "ejectional"[All Fields] OR "ejections"[All Fields] OR "ejects"[All Fields]) AND ("dose fractionation, radiation"[MeSH Terms] OR ("dose"[All Fields] AND "fractionation"[All Fields] AND "radiation"[All Fields]) OR "radiation dose fractionation"[All Fields] OR "fractionation"[All Fields] OR "chemical fractionation"[MeSH Terms] OR ("chemical"[All Fields] AND "fractionation"[All Fields]) OR "chemical fractionation"[All Fields] OR "fraction"[All Fields] OR "fraction s"[All Fields] OR "fractionate"[All Fields] OR "fractionated"[All Fields] OR "fractionates"[All Fields] OR "fractionating"[All Fields] OR "fractionationed"[All Fields] OR "fractionations"[All Fields] OR "fractionator"[All Fields] OR "fractionators"[All Fields] OR "fractioned"[All Fields] OR "fractioning"[All Fields] OR "fractionized"[All Fields] OR "fractions"[All Fields]) | 10794 |
| #3 | #1 OR #2  Filters: Randomized Controlled Trial; Clinical Trial | 825 |

Cochrane Library

| **Query** | **Results** |
| --- | --- |
| (heart failure with preserved ejection fraction) OR (HFpEF)  Filters: Trial | 1662 |

Embase

| **NO.** | **Query** | **Results** |
| --- | --- | --- |
| #1 | 'heart failure with preserved ejection fraction'/exp OR 'heart failure with preserved ejection fraction' | 16471 |
| #2 | 'hfpef' | 12273 |
| #3 | #1 OR #2 | 19765 |
| #4 | #1 OR #2 Filters: Randomized Controlled Trial; Clinical Trial | 1327 |

**Supplementary Table S2: Basic information of the included studies.**

| Study | Sample size of placebo group | Treatment  duration (week) | Age  (year) | BMI  (kg/m^2^) | Female  (%) | LVEF  (%) | White  (%) | 6MWD Baseline (m) | RSD of change from baseline in 6MWD |
| --- | --- | --- | --- | --- | --- | --- | --- | --- | --- |
| S.D. Solomon 2022 | 3132 | 156 | 71.5 | NA | 44.2 | 54.3 | 71 | NA | NA |
| S.D. Anker 2021 | 2991 | 156 | 71.9 | 29.9 | 44.7 | 54.3 | 75.4 | NA | NA |
| Bertram Pitt 2014 | 1723 | 312 | 68.7 | 31 | 51.5 | 56 | 89.2 | NA | NA |
| Salim Yusuf 2003 | 1509 | 182 | 67.1 | 29 | 41 | 54.1 | NA | NA | NA |
| Barry M. Massie 2008 | 2061 | 260 | 72 | 29.7 | 59 | 59 | 94 | NA | NA |
| John G.F. Cleland 2006 | 387 | 52 | 75 | 27.6 | 57 | 64 | NA | 297 | 15.64 |
| Dalane W. Kitzman 2010 | 36 | 52 | 70 | 30 | 89 | 65 | NA | 451.41 | 49.42 |
| Min Zi 2003 | 38 | 26 | 78 | NA | 68.4 | NA | NA | 214.6 | 3.01 |
| Viviane M. Conraads 2012 | 59 | 26 | 34 | 65.3 | 30.2 | 64 | 63.2 | 412 | 5.03 |
| Gavin A. Lewis 2021 | 47 | 52 | 81 | 29 | 45 | 65 | 92 | 262 | 10.1 |
| NCT03448406 | 158 | 12 | 73.9 | NA | 41.8 | NA | 85.4 | 299.5 | 15.0 |
| M.N. Kosiborod 2023 | 232 | 52 | 69 | 36.9 | 55.6 | 57 | 94.7 | 325.8 | 10.8 |
| Theresa-Marie Dachs 2022 | 56 | 26 | 72.1 | 30.3 | 66.1 | 60.1 | 100 | 342.57 | 5.34 |
| NCT03547583 | 262 | 24 | 72.8 | 30.7 | 46.2 | 56.3 | 84.7 | 295.8 | 8.90 |
| John J.V. McMurray 2024 | 251 | 16 | 73 | 28 | 37.1 | 53 | 70.9 | 323 | 12.4 |
| Bharathi Upadhya, MD 2018 | 27 | 26 | 70.6 | 33.7 | 85 | 61 | 52 | 402.64 | 6.08 |
| Bharathi Upadhya, MD 2017 | 38 | 39 | 72 | 32.4 | 79 | 62 | NA | 414.83 | 6.55 |
| NCT03037580 | 43 | 24 | 74 | NA | 72.1 | NA | 83.7 | NA | 7.76 |
| NCT03098979 | 76 | 20 | 73.1 | NA | 47.4 | 57.09 | 84.2 | 322.8 | 273 |
| Michel Komajda 2024 | 84 | 35 | 73 | 28.8 | 67.9 | 61 | NA | 321 | 3.74 |
| Mayo Clinic 2014 | 103 | 24 | 69 | 32.8 | 53 | 60 | 90 | 305 | 3.51 |
| M.N. Kosiborod 2024 | 306 | 52 | 70 | 36.9 | 47.4 | 55 | 87.6 | 280 | NA |
| NCT03254485 | 90 | 12 | 70.1 | NA | 44.4 | NA | 72.2 | NA | 4.68 |
| NCT02713126 | 36 | 12 | 73.5 | NA | 55.6 | NA | 100 | NA | 3.01 |
| Janet D. Pierce, PhD 2022 | 38 | 12 | 69 | NA | 59 | 51.04 | NA | 247.87 | 3.89 |
| ANITA DESWAL 2011 | 23 | 26 | 68.7 | 34.6 | 8.7 | 62.5 | NA | 249 | 2.53 |
| Michael E. Nassif 2021 | 162 | 12 | 71 | 34.6 | 56.8 | 60 | 69 | 244 | 60.9 |
| Eduard Shantsila 2020 | 106 | 104 | 72 | 30 | 25 | NA | 94 | 271 | 2.50 |
| Frank Edelmann, MD 2013 | 209 | 52 | 67 | 28.9 | 53 | 68 | NA | 531 | 17.7 |

NA: not reported; RSD: Relative Standard Deviation.

The RSD shown in the table are the end points of the trial

The above data should prioritize entering the mean; if the study does not provide the mean, the median should be entered instead.

**Supplementary Table S3:** **The list of included studies.**

| ID | Author/NCT Number | Publication Year  /Last Update Year | Research Title |
| --- | --- | --- | --- |
| 1 | S.D. Solomon | 2022 | Dapagliflozin in Heart Failure with Mildly Reduced or Preserved Ejection Fraction |
| 2 | S.D. Anker | 2021 | Empagliflozin in Heart Failure with a Preserved Ejection Fraction |
| 3 | Bertram Pitt | 2014 | Spironolactone for Heart Failure with Preserved Ejection Fraction |
| 4 | Salim Yusuf | 2003 | Effects of candesartan in patients with chronic heart failure and preserved left-ventricular ejection fraction: the CHARM-Preserved Trial |
| 5 | Barry M. Massie | 2008 | Irbesartan in Patients with Heart Failure and Preserved Ejection Fraction |
| 6 | John G.F. Cleland | 2006 | The perindopril in elderly people with chronic heart failure (PEP-CHF) study |
| 7 | Dalane W. Kitzman | 2010 | A Randomized Double-Blind Trial of Enalapril in Older Patients With Heart Failure and Preserved Ejection Fraction Effects on Exercise Tolerance and Arterial Distensibility |
| 8 | Min Zi | 2003 | The Effect of Quinapril on Functional Status of Elderly Patients with Diastolic Heart Failure |
| 9 | Viviane M. Conraads | 2012 | Effects of the long-term administration of nebivolol on the clinical symptoms, exercise capacity, and left ventricular function of patients with diastolic dysfunction: results of the ELANDD study |
| 10 | Gavin A. Lewis | 2021 | Pirfenidone in heart failure with preserved ejection fraction: a randomized phase 2 trial |
| 11 | NCT03448406 | 2020 | This Study Tests Empagliflozin in Patients With Chronic Heart Failure With Preserved Ejection Fraction (HFpEF). The Study Looks at How Far Patients Can Walk in 6 Minutes and at Their Heart Failure Symptoms. |
| 12 | M.N. Kosiborod | 2023 | Semaglutide in Patients with Heart Failure with Preserved Ejection Fraction and Obesity |
| 13 | Theresa-Marie Dachs | 2022 | Riociguat in pulmonary hypertension and heart failure with preserved ejection fraction: the haemoDYNAMIC trial |
| 14 | NCT03547583 | 2021 | Patient-reported Outcomes in Vericiguat-treated Patients With HFpEF (VITALITY-HFpEF) |
| 15 | John J.V. McMurray | 2024 | Effect of Dapagliflozin Versus Placebo on Symptoms and 6-Minute Walk Distance in Patients With Heart Failure: The DETERMINE Randomized Clinical Trials |
| 16 | Bharathi Upadhya, MD | 2018 | The Effect of Aliskiren on Exercise Capacity in Older Patients with Heart Failure and Preserved Ejection Fraction: A Randomized, Placebo-Controlled, Double-Blind Trial |
| 17 | Bharathi Upadhya, MD | 2017 | The Effect of Spironolactone on Exercise Tolerance and Arterial Function in Older Patients with HEpEF |
| 18 | NCT03037580 | 2020 | Oral Treprostinil in Subjects With Pulmonary Hypertension Associated With Heart Failure With Preserved Ejection Fraction |
| 19 | NCT03098979 | 2019 | A Trial to Study Neladenoson Bialanate Over 20 Weeks in Patients With Chronic Heart Failure With Preserved Ejection Fraction (PANACHE) |
| 20 | Michel Komajda | 2017 | Effect of ivabradine in patients with heart failure with preserved ejection fraction: the EDIFY randomized placebo-controlled trial |
| 21 | Mayo Clinic | 2014 | Effect of Phosphodiesterase-5 Inhibition on Exercise Capacity and Clinical Status in Heart Failure with Preserved Ejection Fraction: A Randomized Clinical Trial |
| 22 | M.N. Kosiborod | 2024 | Semaglutide in Patients with Obesity-Related Heart Failure and Type 2 Diabetes |
| 23 | NCT03254485 | 2022 | A Study of the Effect of IW-1973 on the Exercise Capacity of Patients With Heart Failure With Preserved Ejection Fraction (HFpEF) (CAPACITY-HFpEF) |
| 24 | NCT03756285 | 2021 | Safety and Tolerability Study of AZD4831 in Patients With Heart Failure. (SATELLITE) |
| 25 | Janet D. Pierce, PhD | 2022 | Effects of Ubiquinol and/or D-ribose in Patients With Heart Failure With Preserved Ejection Fraction |
| 26 | ANITA DESWAL | 2011 | Results of the Randomized Aldosterone Antagonism in Heart Failure With Preserved Ejection Fraction Trial (RAAM-PEF) |
| 27 | Michael E. Nassif | 2021 | The SGLT2 inhibitor dapagliflozin in heart failure with preserved ejection fraction: a multicenter randomized trial |
| 28 | Eduard Shantsila | 2020 | Spironolactone in Atrial Fibrillation With Preserved Cardiac Fraction: The IMPRESS-AF Trial |
| 29 | Frank Edelmann, MD | 2013 | Effect of Spironolactone on Diastolic Function and Exercise Capacity in Patients With Heart Failure With Preserved Ejection Fraction The Aldo-DHF Randomized Controlled Trial |

**Supplementary Figure S1: The feature map of the included studies.**

| 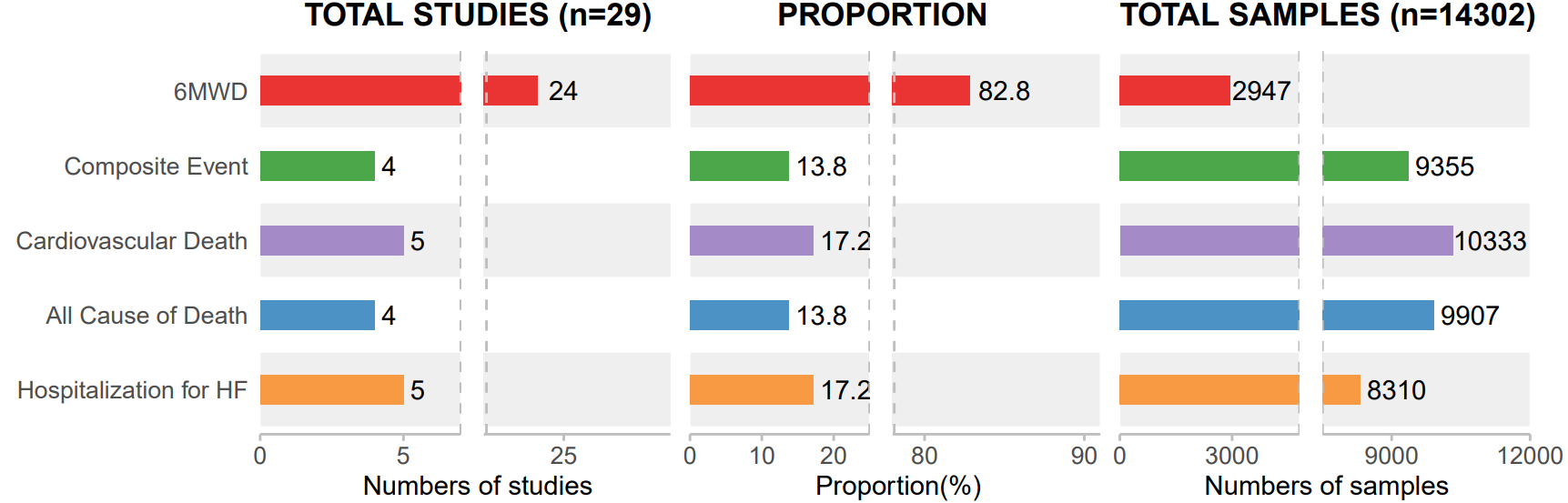 |
| --- |
| “6MWD” represents change from baseline in 6-minute-walk-distance,  “Composite Event” represents cardiovascular or hospitalization for heart failure,  “HF” represents heart failure. |

**Supplementary Table S4. The results of the literature assessment.**

| **Study** | **Randomization process** | **Deviations from intended interventions** | **Missing outcome data** | **Measurement of the outcome** | **Selection of the reported result** | **Overall Bias** |
| --- | --- | --- | --- | --- | --- | --- |
| S.D. Solomon 2022 | Low | Low | Some concerns | Low | Low | Some concerns |
| S.D. Anker 2021 | Low | Low | Some concerns | Low | Low | Some concerns |
| Bertram Pitt 2014 | Low | Low | High | Low | Low | High |
| Salim Yusuf 2003 | Low | Low | High | Some concerns | Low | High |
| Barry M. Massie 2008 | Low | Low | Some concerns | Low | Low | Some concerns |
| John G.F. Cleland 2006 | Low | Low | Some concerns | Low | Low | Some concerns |
| Dalane W. Kitzman 2010 | Low | Low | Low | Low | Low | Low |
| Min Zi 2003 | Some concerns | Low | High | Some concerns | Low | High |
| Viviane M. Conraads 2012 | Low | Low | Some concerns | Low | Low | Some concerns |
| Gavin A. Lewis 2021 | Low | Low | Some concerns | Low | Low | Some concerns |
| NCT03448406 | Low | Some concerns | High | Low | Some concerns | High |
| M.N. Kosiborod 2023 | Low | Low | Some concerns | Low | Low | Some concerns |
| Theresa-Marie Dachs 2022 | Low | High | Some concerns | Low | Low | High |
| NCT03547583 | Low | Low | Some concerns | Low | Low | Some concerns |
| John J.V. McMurray 2024 | Low | High | High | Low | Low | High |
| Bharathi Upadhya, MD 2018 | Low | Low | Some concerns | Low | Low | Some concerns |
| Bharathi Upadhya, MD 2017 | Low | Some concerns | High | Low | Low | High |
| NCT03037580 | Low | Low | Low | Low | Low | Low |
| NCT03098979 | Low | High | Some concerns | Low | Low | High |
| Michel Komajda 2024 | Low | Low | Some concerns | Low | Low | Some concerns |
| Mayo Clinic 2014 | Low | High | High | Some concerns | Low | High |
| M.N. Kosiborod 2024 | Low | Some concerns | Some concerns | Low | Low | Some concerns |
| NCT03254485 | Low | Some concerns | Low | Low | Low | Some concerns |
| NCT02713126 | Low | Some concerns | High | Low | Low | High |
| Janet D. Pierce, PhD 2022 | Low | Low | Low | Low | Low | Low |
| ANITA DESWAL 2011 | Low | High | Low | Some concerns | Low | High |
| Michael E. Nassif 2021 | Low | Low | Low | Low | Low | Low |
| Eduard Shantsila 2020 | Low | Low | Low | Some concerns | Low | Some concerns |
| Frank Edelmann, MD 2013 | Low | Low | Low | Low | Low | Low |

**Supplementary Figure S2. Risk of bias assessment chart.**

|  | **Cardiovascular death or**  **hospitalized for heart failure** | | **Cardiovascular death** | | **All cause of death** | | **Hospitalized for heart failure** | |
| --- | --- | --- | --- | --- | --- | --- | --- | --- |
|  | **Estimates**  **(RSE,%)** | **SIR Median**  **(95%CI)** | **Estimates**  **(RSE,%)** | **SIR Median**  **(95%CI)** | **Estimates**  **(RSE,%)** | **SIR Median**  **(95%CI)** | **Estimates**  **(RSE,%)** | **SIR Median**  **(95%CI)** |
| **Parameters** | | |  |  |  |  |  |  |
| SIGM | 2.28  (1.20) | 2.28  (2.24, 2.33) | 1.88  (2.20) | 1.88  (1.80, 1.94) | 1.51  (2.60) | 1.50  (1.44, 1.57) | 2.44  (12.5) | 2.44  (2.21, 2.69) |
| MU | 5.39  (1.40) | 5.39  (5.26, 5.53) | 6.05  (1.00) | 6.03  (5.92, 6.13) | 5.08  (2.60) | 5.07  (4.87, 5.25) | 5.75  (8.00) | 5.77  (5.24, 6.29) |
| **Variability** |  |  |  |  |  |  |  |  |
| ηMU | 0.0260  (11.6) | 0.0262  (0.0209, 0.0312) | 0.00800  (33.4) | 0.00914  (0.00466, 0.0132) | 0.0350  (16.4) | 0.0354  (0.0232, 0.0452) | 0.0520  (28.0) | 0.0529  (0.0303, 0.0728) |
| ε | 0.0465  (8.10) | 0.473  (0.394, 0.540) | 0.808  (8.40) | 0.812  (0.687, 0.940) | 1.25  (13.1) | 1.27  (1.02, 1.51) | 1.56  (4.90) | 1.55  (1.44, 1.67) |

**Supplementary Table S5：Estimated parameters for the placebo response model.**

|  | **Change from baseline in 6MWD** | | |
| --- | --- | --- | --- |
|  | **Estimates** | **RSE, %** | **SIR Median (95%CI)** |
| **Parameters** | | | |
| B_0_ | 0.357 | 32.5 | 0.357(0.174,0.580) |
| **Variability** | | | |
| ηB_0_ | 0.779 | 25.8 | 0.772 (0.318,1.11) |
| ε | 9.607 | 22.0 | 10.1(6.15,13.2) |

| **Supplementary Figure S3. The goodness plots of the final model.** |
| --- |
| **Cardiovascular death or hospitalized for heart failure** |
| 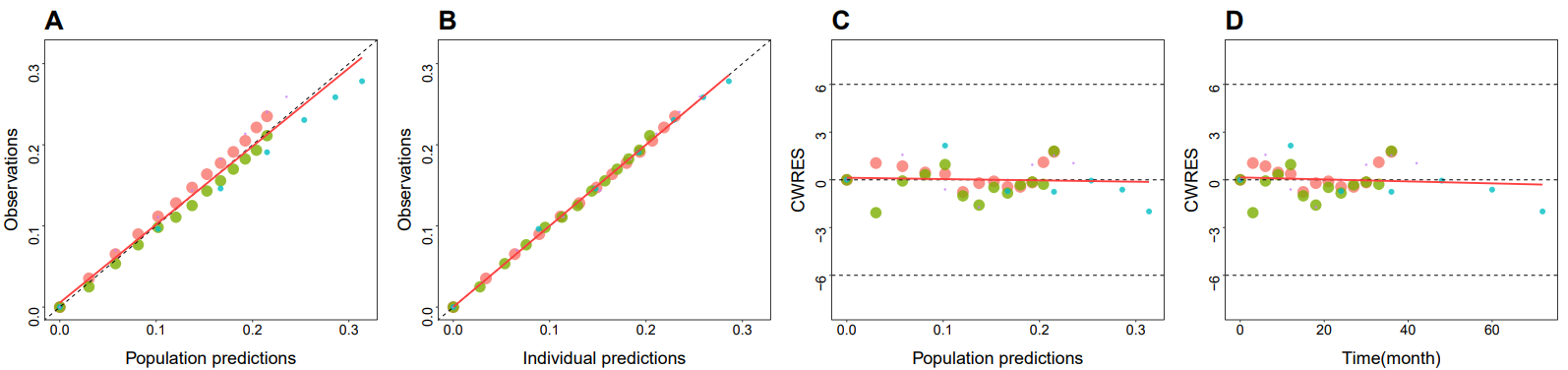 |
| **Cardiovascular death** |
| 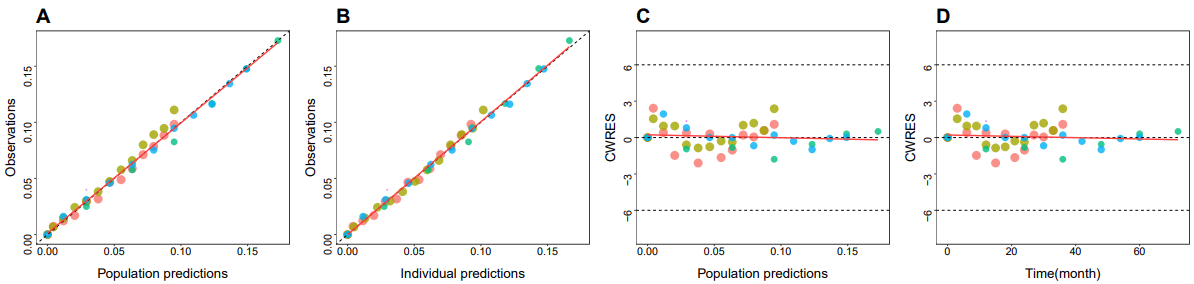 |
| **All cause of death** |
| 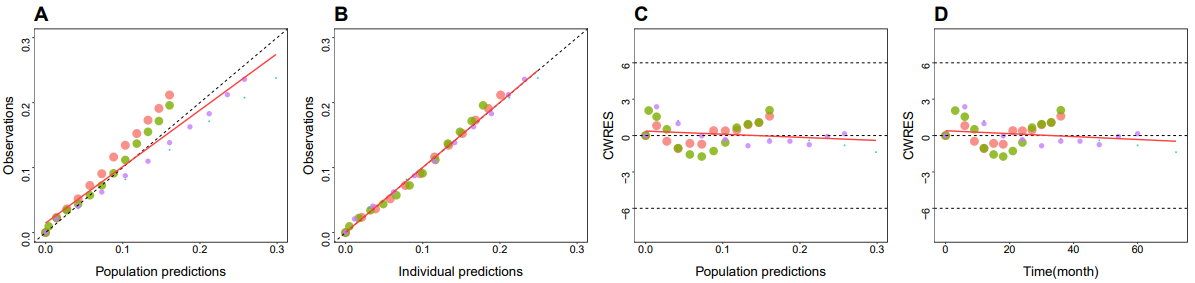 |
| **Hospitalized for heart failure** |
| 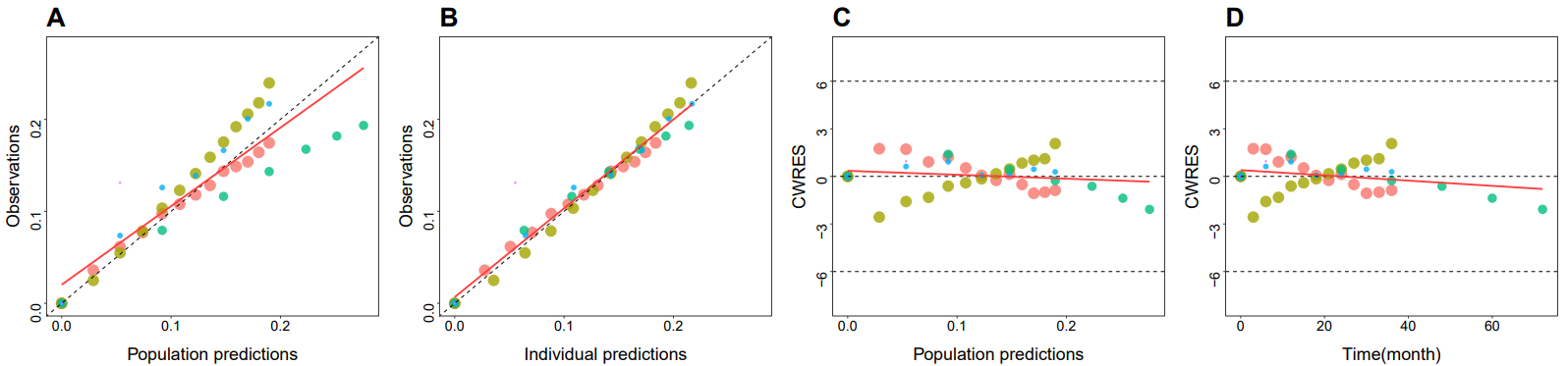 |
| **Change from baseline in 6MWD** |
| 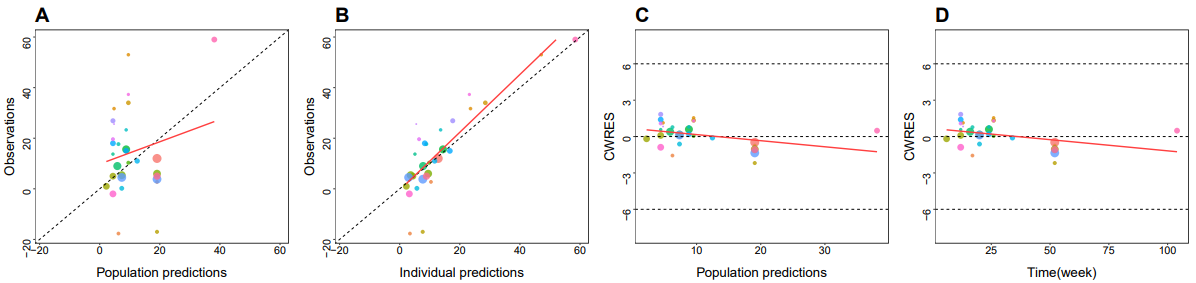 |
| Red solid lines represent the regression lines. The black dashed lines in the figure A and B are the diagonal reference line. The black dashed lines in the C and D are the position where CWRES equal 0 and ± 6. CWRES = conditional weighted residuals. The fitted regression line depicted in the figure has been derived using the method of ordinary least squares. |

| **Supplementary Table S6: Basic information of HFpEF clinical trials with event incidence as the primary outcome measure.** | | | | |  |
| --- | --- | --- | --- | --- | --- |
| Trial/Author, year | Sample size of placebo group | Agent | Accrual time  (months) | Follow-up time  (months) | |
| EMPEROR-PRESERVED | 2991 | Empagliflozin | 36.5 | 26.2 | |
| I-PRESERVED | 2061 | Irbesartan | 34 | 49.5 | |
| DELIVER | 3132 | Dapagliflozin | 28 | 27.6 | |
| TOPCAT | 1723 | Spironolactone | 65.6 | 39.6 | |
| CHARM-PRESERVED | 3032 | Candesartan | 16 | 36.6 | |
| John G.F.Cleland, 2006 | 424 | Perindopril | 36 | 25.2 | |
| Min Zi, 2003 | 38 | Quinapril | 24 | 6 | |

| **Supplementary Table S7: The definition of composite outcome events included in the trials.** | |
| --- | --- |
| **Trial** | **Composite outcome Event Definition** |
| DELIVER | Composite endpoint of heart failure worsening or cardiovascular death. |
| EMPEROR-PRESERVED | Cardiovascular death or hospitalization for heart failure. |
| TOPCAT | Composite endpoint of cardiovascular death, myocardial infarction, or hospitalization for heart failure. |
| CHARM-PRESERVED | Cardiovascular death or hospitalization for congestive heart failure. |

| **Supplementary Table S8：The results of the sensitivity analysis of the composite outcome indicators** | | |  |
| --- | --- | --- | --- |
|  | **Original model** | **Model of removing the DELIVER** | |
|  | **Estimates (RSE, %)** | **Estimates (RSE, %)** | |
| SIGM | 2.28 (1.20) | 2.27 (1.80) | |
| MU | 5.39 (1.40) | 5.41 (1.90) | |
| ηMU | 0.0260 (11.6) | 0.0260 (20.5) | |
| ε | 0.0465 (8.10) | 0.508 (5.60) | |

| **Supplementary Figure S4: Sample size estimation results under different conditions (taking cardiovascular death or hospitalized for heart failure as an example)** | | |
| --- | --- | --- |
| Power=0.8, HR=0.7 | Power =0.8, HR=0.75 | Power =0.8, HR=0.8 |
| 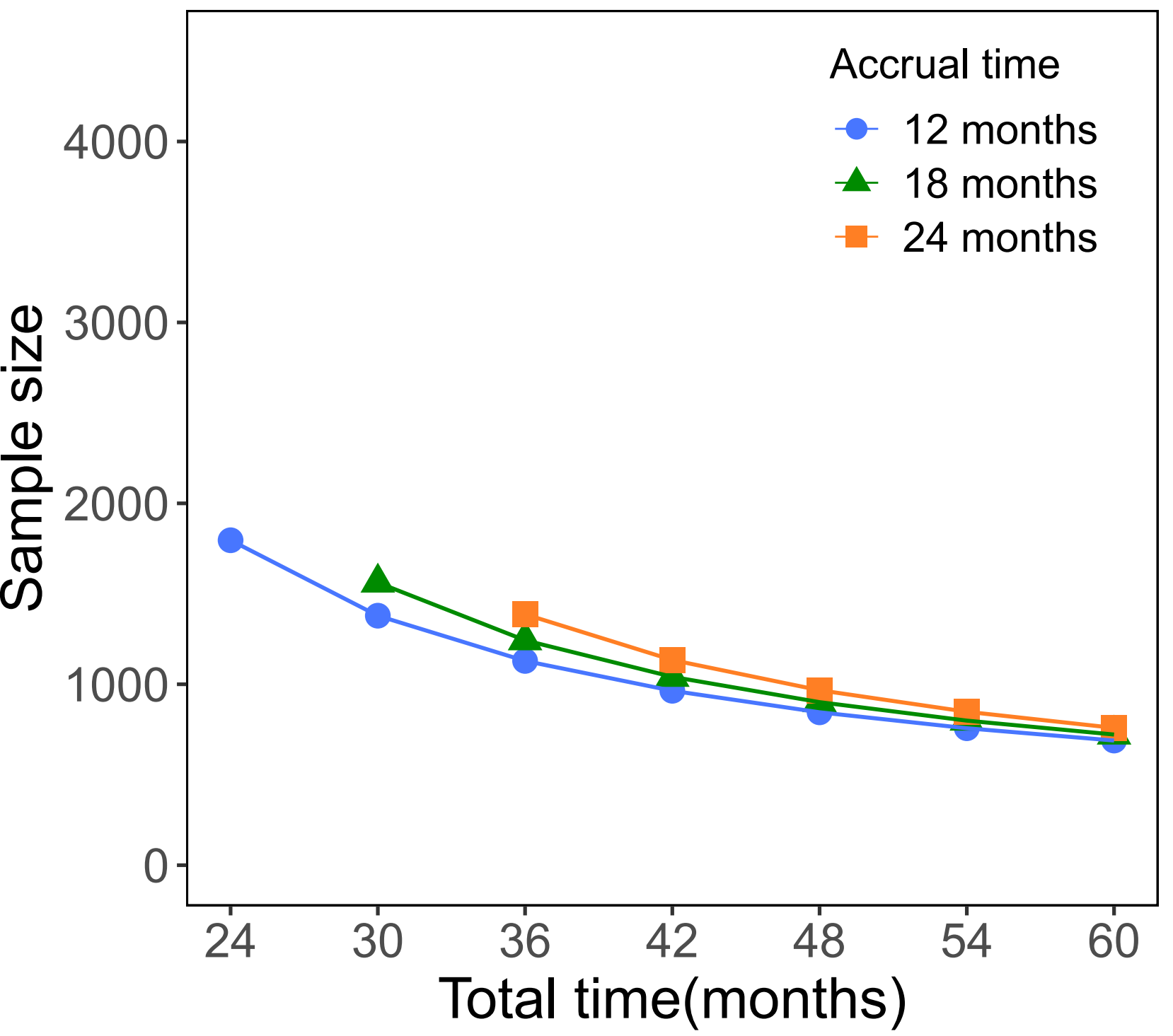 | 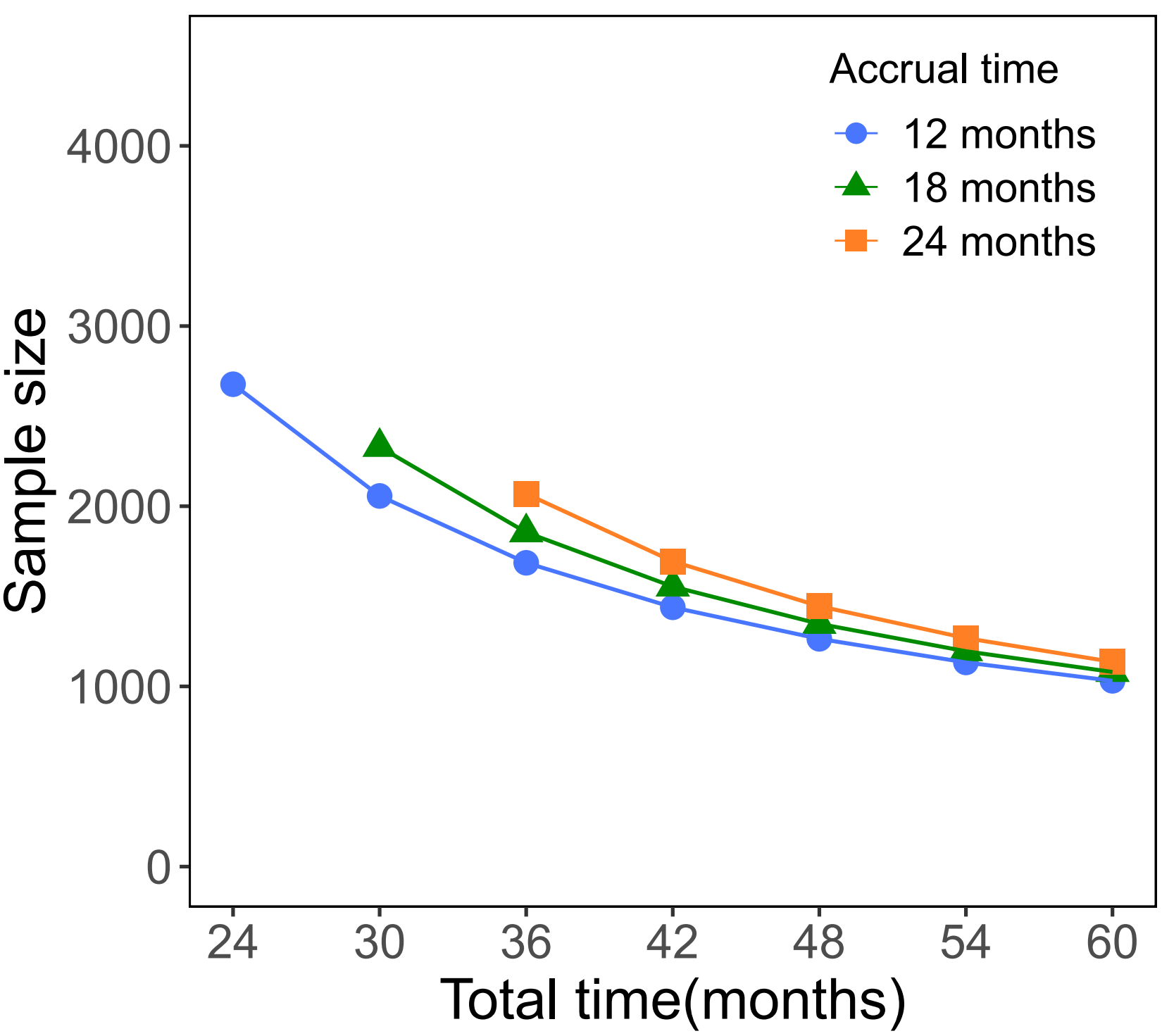 | 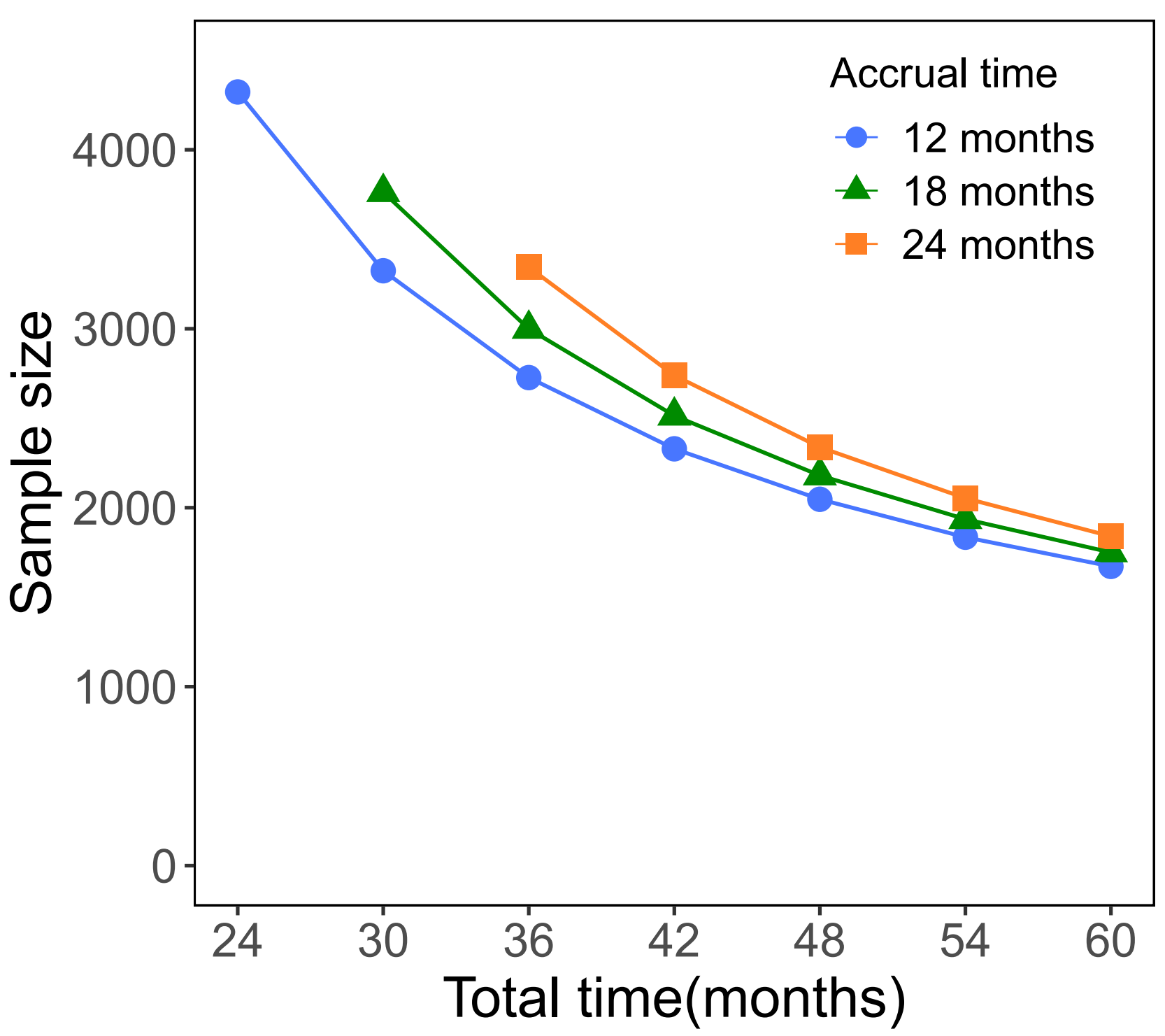 |
| Power=0.9, HR=0.7 | Power=0.9, HR=0.75 | Power=0.9, HR=0.8 |
| 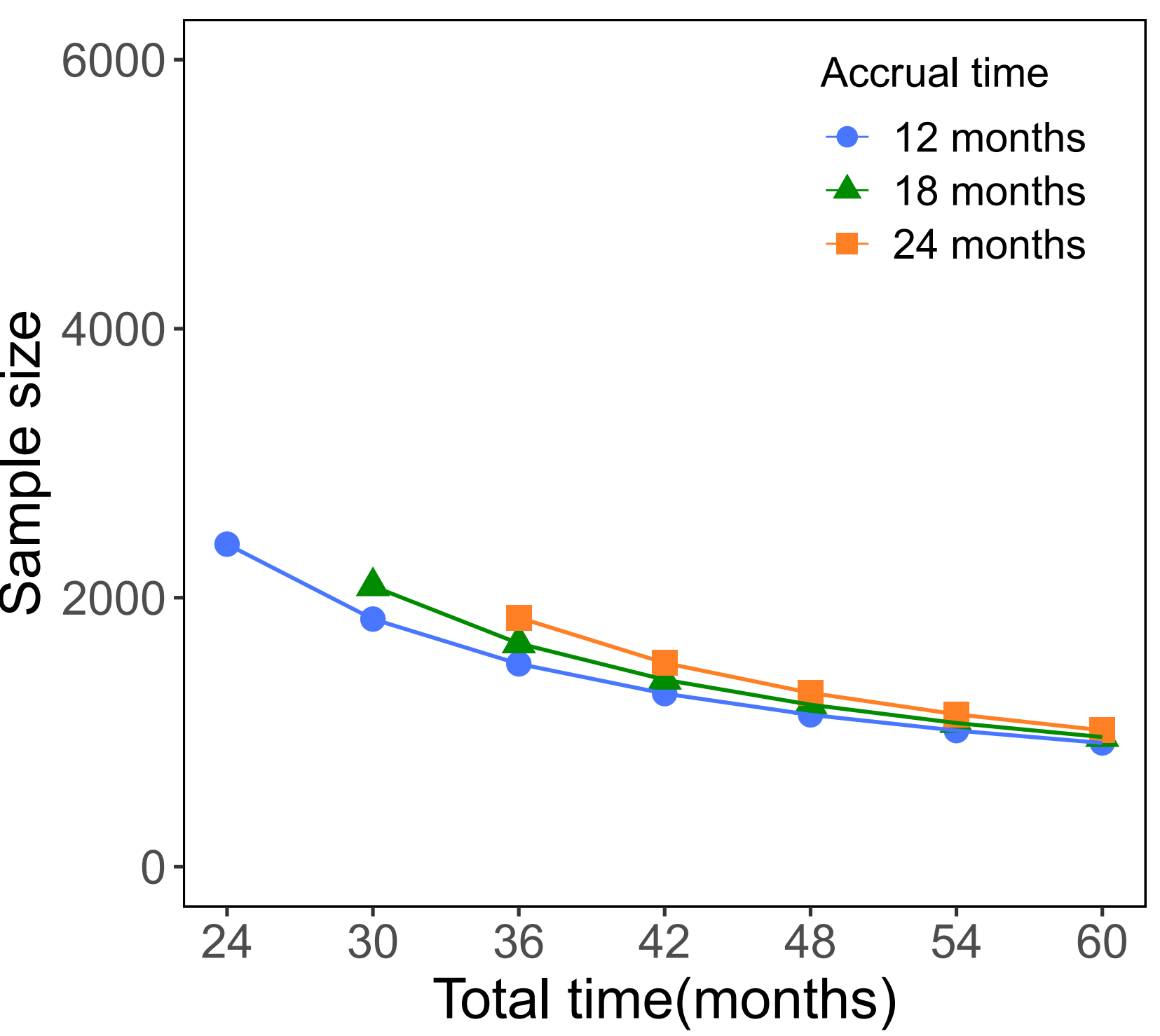 | 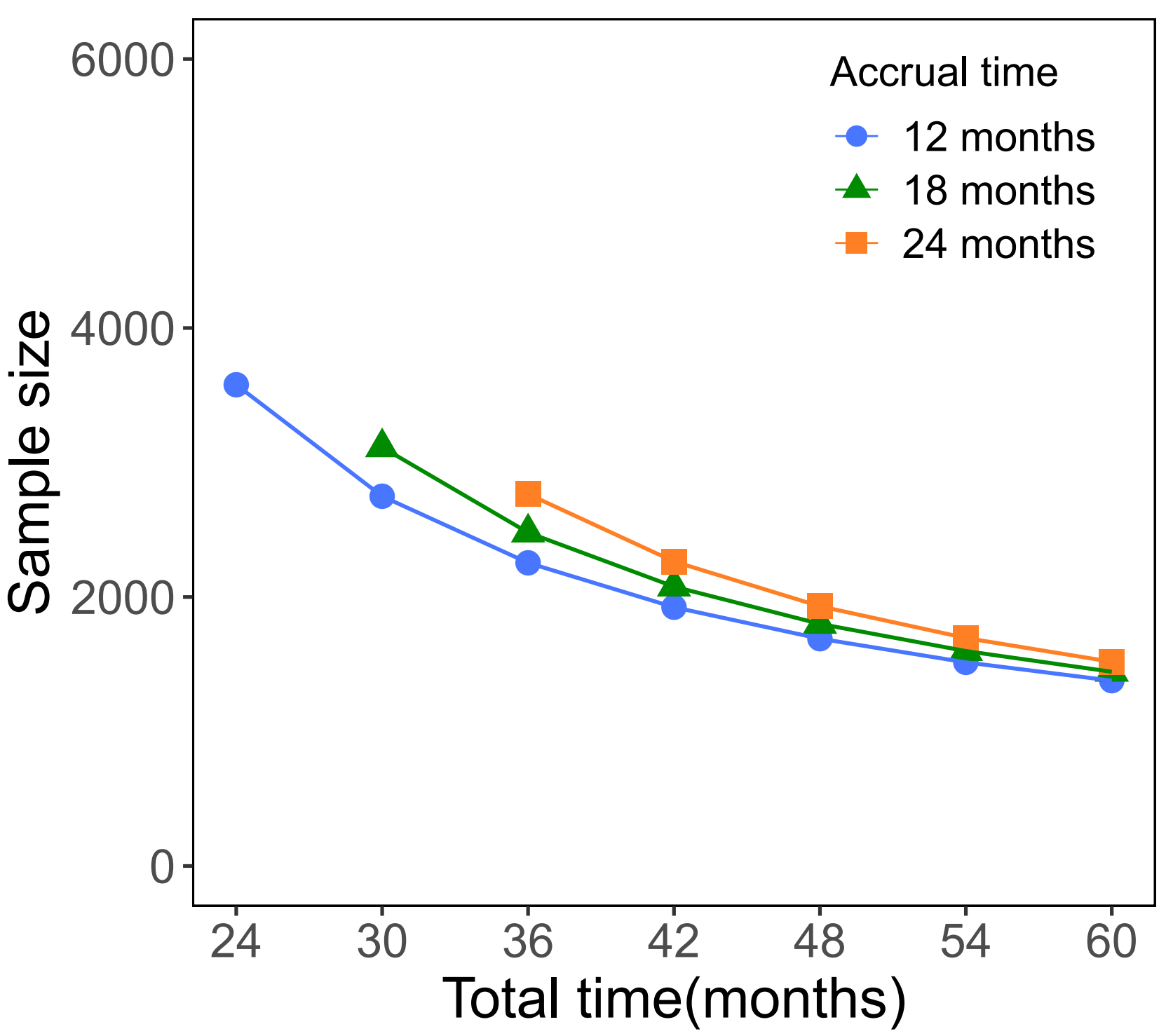 | 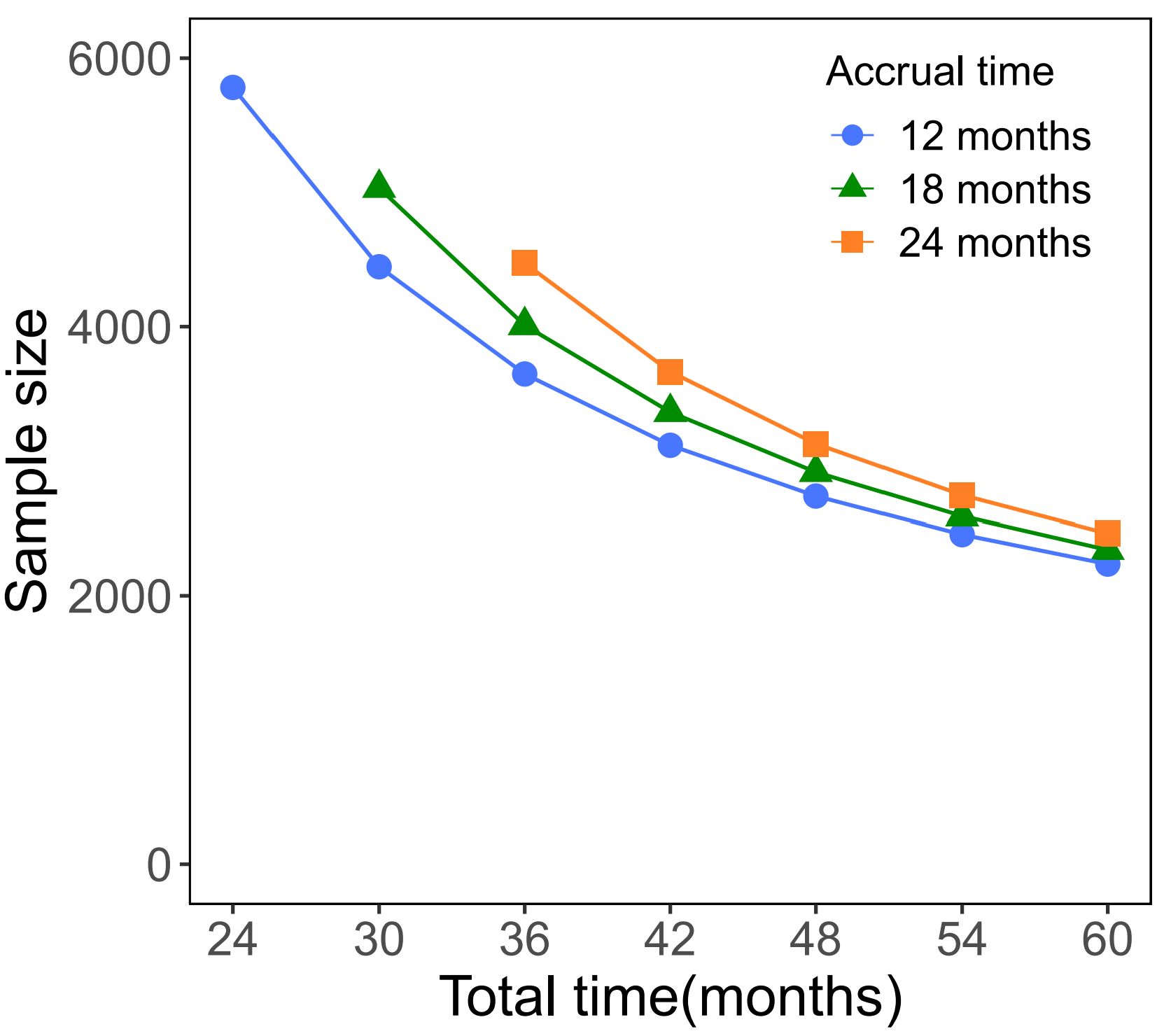 |
| In all scenarios, the incident rate is set to 1-year typical value 0.111, the significance level (α) is set to 0.05, the drug group and the placebo group were randomly assigned at 1:1. | | |

|  |  |
| --- | --- |

**Supplementary** **Methods S1: Inclusion and Exclusion Criteria** **and Data Extraction**

**The inclusion criteria were as follows:**

(1) randomized, double-blind, placebo-controlled trials;

(2) patients with a LVEF of no less than 50%, or those diagnosed with heart failure with preserved ejection fraction as defined by the researchers;

(3) the trial must report at least one of the following outcome measures: cardiovascular mortality, hospitalization rates due to heart failure, rates of cardiovascular death, all-cause mortality, hospitalization due to heart failure, or changes in 6MWD from baseline.

**The exclusion criteria were as follows:**

(1) conference abstracts, which are typically not peer-reviewed and lack comprehensive information;

(2) non-original data publications such as meta-analyses and reviews, which were excluded to prevent the duplication of data utilization.

**Data extraction method:**

An Excel-based data entry template was employed to extract information from literature that met the inclusion criteria. The data extracted included bibliographic characteristics (literature ID, authors, publication year, and country), trial design (trial groups, intervention groups, dosing regimens, sample size, duration of treatment, etc.), participant characteristics (age, BMI, heart rate per minute, percentage of female participants, percentage of Caucasian participants, baseline LVEF, percentage of participants with diabetes, percentage of participants with hypertension, percentage of participants with a history of heart failure hospitalization, patients under each New York Heart Association (NYHA) Classification, and baseline 6MWD), and outcomes (event rates and 6MWD at each follow-up point). All information was independently extracted by two researchers (X.C. and J.Y.), with any discrepancies resolved by a third investigator (L.L.).

**Supplementary Methods S2: Model building**

This study aims to develop a time-effect model for the placebo group, capturing cardiovascular death or hospitalized for heart failure, cardiovascular death, all cause of death, hospitalized for heart failure and changes from baseline in 6MWD. Models building included the establishment of structural, random effect and covariate models.

In terms of the structural model, survival models will be used to model cardiovascular death or hospitalized for heart failure, cardiovascular death, all cause of death, and hospitalized for heart failure. A linear model will describe the trend of changes from baseline in 6MWD, which is expected to show a linear increase over time.

For the random effects model, inter-trial variability will be examined using additive and exponential models, while residual errors will be explored through additive, proportional, and proportional plus additive models.

In the covariate model, this study will investigate the following influencing factors: age, BMI, heart rate per minute, the proportion of female participants, the proportion of Caucasian participants, and LVEF values. Detailed modeling specifics are as followed.

Survival model was used to model cardiovascular death or hospitalized for heart failure rate, all cause of death, cardiovascular death, hospitalized for heart failure rate. The survival model is related to the risk function h(t), which can be interpreted as the instantaneous risk of death at a point in time t. Formula 1 describes the relationship between survival function and risk function:

Survival model $S(t)=exp(-\int_{0}^{t} h(t)dt)$ (1)

In Formula 1，$\int_{0}^{t} h(t)dt$ is the cumulative risk from point 0 to point t，S(t) is the survival rate at time t. Through this formula, the risk function can be transformed into a survival function.

This study will examine four different risk function models（Formula 2-5）：

Constant: $h\left( t \right)=\lambda$ (2)

Gompert: $h\left( t \right)=\lambda\cdot exp(\beta\cdot t)$ (3)

Weibull: $h\left( t \right)=\lambda\cdot exp(\beta\cdot Ln(t))$ (4)

Lognormal: $h\left( t \right)=\frac{{(\sigma t\sqrt{2\pi})}^{-1}e^{{(-\frac{1}{2}z}^{2})}}{1-\emptyset(z)}, Z=\frac{\ln\left( t \right)-\mu}{\sigma}$ (5)

The risk function in Formulas 2-4 is defined by parameters λ and β, which represent the mortality risk rate at time 0 and the rate at which the mortality risk rate changes over time, respectively. The risk function in Formula 5 is defined by the parameters μ and σ, which represent the median and standard deviation of the lognormal distribution, respectively.

Data exploration analysis showed that the change from baseline in 6MWD showed a linear increase trend over time, and this data feature could be described by a linear model (Formula 7) :

$E=-B0\times time+B1$ (7)

In Formula 7, E represents the efficacy of the placebo group on 6MWD at the time time point, B0 represents the slope, B1 represents the intercept, and time is the observation time in weeks.

In order to describe the difference between the observed value and the predicted value of the model, inter-trial variation and residual variation need to be included in the model. The inter-trial variation will look at the addition model (Formula 8) or the exponential model (Formula 9), and the residual variation will look at the addition model (Formula 10), the scale model (Formula 11), and the scale addition model (Formula 12). According to the objective function value (OFV) estimated by NONMEM software, the relative standard error (RSE) of the model parameter estimates and the goodness-of-fit (GOF) graph, the better model is selected.

$P_{i}=P_{\text{pop}}+\eta_{i}$ (8)

$P_{i}=P_{\text{pop}}\times\exp(\eta_{i})$ (9)


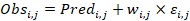
$\mathrm{Obs}_{i,j}=\mathrm{Pred}_{i,j}+w_{i,j}\times{\varepsilon1}_{i,j}$ (10)


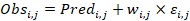
$\mathrm{Obs}_{i,j}=\mathrm{Pred}_{i,j}\times(1+w_{i,j}\times{\varepsilon2}_{i,j})$ (11)


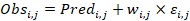
$\mathrm{Obs}_{i,j}=\mathrm{Pred}_{i,j}\times(1+w_{i,j}\times{\varepsilon2}_{i,j})+w_{i,j}\times{\varepsilon1}_{i,j}$ (12)

$w_{\text{i,j}}\text{= S}\text{E}_{\text{i,j}}=\frac{SD_{i,j}}{\sqrt{{N_{i}}_{,j}}}$ (13)

In Formulas 8-9, Pi、Ppop and ηi are individual test values, population typical values and inter-test variation of model parameters respectively, ηi conforms to the normal distribution with mean 0 and variance ωi2. In Formula 10-12, Obsi,j represent the observed efficacy value of the i test at the j observation time point, and Predi,j represent the predicted value of the i test and j observation time point. Ɛ1i,j is the sum-type residual variation at the jth observation time point of the i experimental group, Ɛ2i,j is the proportional residual variation at the jth observation time point of the i experimental group, conforming to normal distributions with mean 0 and variance σ12and σ22, respectively. In Formula 13, wi,j is the correction factors of the residual error, and the correction is carried out by the reciprocal of the square root of the sample size, that is, it is believed that the larger the sample size, the smaller the residual variation.

The factors affecting model parameters are analyzed by constructing covariate model. These factors included age, BMI, heart beats per minute, percentage of female subjects, percentage of white subjects, and LVEF scores. If the missing rate of a covariate is 30% or more, it is not included in the analysis. If the missing rate is less than 30%, the missing data will be processed using a multi-fill method. In this process, model parameters and missing covariates are put together, Predictive Mean Matching (PMM) is selected as the main filling algorithm, and six rounds of filling are performed. In order to obtain the final data analysis results, the calculation results of these 6 times will be integrated.

For a model of event incidence, covariates are added to the underlying risk function in the following form:

$h(t){\_}_{\mathrm{Final}}=h(t){\_}_{\mathrm{Base}}\cdot e^{(\alpha_{1}\cdot x_{1}+\alpha_{2}\cdot x_{2}+\cdots+\alpha_{n}\cdot x_{n})}$
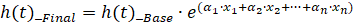
 (14)

In Formula 14,
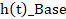
$h(t){\_}_{\mathrm{Base}}$ is the basic risk function，$e^{(\alpha_{1}\cdot x_{1}+\alpha_{2}\cdot x_{2}+\cdots+\alpha_{n}\cdot x_{n})}$
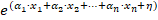
 describes the effect of covariates on the risk of death, where x1, x2, …, xn represents 1-n covariates that have an impact on the risk of death, and their influence coefficients on the risk of death are α1, α2, …, αn.

For the model with change from the baseline in 6MWD, the introduction of continuous variables is shown in Formula 15-16, and the introduction of binary variables is shown in Formula 17:

$P_{i}=P_{typical}+(COVi-COVmedian)\times\theta COV$ (15)

$P_{i}=P_{typical}+(COVi/COVmedian)^{\theta COV}$ (16)

$P_{i}=P_{typical}+COVi\times\theta COV$ (17)

In Formulas 15-17, Pi represents the individual value of the ith test model parameter, COVmedian is the median of covariables in the data set, Ptypical represents the group typical value of the model parameter when the classification covariable is equal to 0 or the continuous covariable is equal to COVmedian, COVi represents the covariable value of the ith test. θcov is the correction factor of the covariable for the model parameters.

The covariates were screened by forward introduction and reverse elimination methods. In the forward introduction phase, the threshold of OFV was set to 3.84 (P < 0.05). In the reverse elimination stage, the threshold of OFV was set to 6.63 (P < 0.01).

**Supplementary Methods S3: Model Assessment Method**

Upon completion of the model development, the goodness-of-fit will be evaluated using diagnostic plots, which include comparisons between observed values (OBS) and population predictions (PRED), observed values (OBS) and individual predictions (IPRED), conditional weighted residuals (CWRES) versus PRED, and scatter plots of CWRES against time.

Additionally, visual predictive checks (VPC) were employed to evaluate the predictive performance of the model. This involved obtaining 90% confidence intervals for the pharmacodynamic values of the placebo group at different time points through 1,000 Monte Carlo simulations, and comparing these intervals with the actual observed data. If the majority of the observed pharmacodynamic values fall within the confidence intervals, it indicates that the model has good predictive performance.

The robustness of the model will be evaluated using Sampling Importance Resampling (SIR). If the parameter estimates obtained by the SIR method are close to those of the final model, it suggests that the model's parameter estimates are robust and minimally influenced by individual studies.

**Supplementary Methods S4: Model NONMEM Codes**

**NONMEN codes of cardiovascular death or hospitalized for heart failure model:**

$PROBLEM EFFECT

$DATA CD-HHF.csv IGNORE=C

$INPUT ID TIME DV SIZE MDV

$SUBROUTINE ADVAN6 TOL=4

$MODEL COMP=(HZLAST)

$PK

SIGM=THETA(1)+ETA(1)

MU=THETA(2)*EXP(ETA(2))

$DES

DEL= 1E-12

TIM=T+DEL

LNT = LOG(TIM)

X1 =(LNT-MU)/SIGM

PDF= EXP(-1/2*(X1**2))/SQRT(2*3.14159265)

DADT(1)=1/(TIM*SIGM)*PDF/(1-PHI(X1))

$ERROR

CUMEVT=A(1)

EFT=1-EXP(-CUMEVT)

IPRED=EFT

W=SQRT((DV+0.001)*(1-DV+0.001)/SIZE)

Y=EFT+W*ERR(1)

$THETA (0,3) ;SIGM

$THETA (0,7) ;MU

$OMEGA 0,FIXED

$OMEGA 0.09

$SIGMA 1

$ESTIMATION METHOD=1 INTER MAXEVAL=9990 PRINT=10 NOHABORT NOTHETABOUNDTEST NOOMEGABOUNDTEST NOSIGMABOUNDTEST POSTHOC

$COVARIANCE

$TABLE ID TIME DV SIZE MDV Y SIGM MU ETA(1) ETA(2) CUMEVT CWRES IPRED NOPRINT ONEHEADER FILE=CD-HHF.txt

**NONMEN codes of cardiovascular death model:**

$PROBLEM EFFECT

$DATA CD.csv IGNORE=C

$INPUT ID TIME DV SIZE MDV

$SUBROUTINE ADVAN6 TOL=4

$MODEL COMP=(HZLAST)

$PK

SIGM=THETA(1)*EXP(ETA(1))

MU=THETA(2)*EXP(ETA(2))

$DES

DEL= 1E-12

TIM=T+DEL

LNT = LOG(TIM)

X1 =(LNT-MU)/SIGM

PDF= EXP(-1/2*(X1**2))/SQRT(2*3.14159265)

DADT(1)=1/(TIM*SIGM)*PDF/(1-PHI(X1))

$ERROR

CUMEVT=A(1)

EFT=1-EXP(-CUMEVT)

IPRED=EFT

W=SQRT((DV+0.001)*(1-DV+0.001)/SIZE)

Y=EFT+W*ERR(1)

$THETA (0,3) ;SIGM

$THETA (0,7) ;MU

$OMEGA 0,FIXED

$OMEGA 0.09

$SIGMA 1

$ESTIMATION METHOD=1 INTER MAXEVAL=9990 PRINT=10 NOHABORT NOTHETABOUNDTEST NOOMEGABOUNDTEST NOSIGMABOUNDTEST POSTHOC

$COVARIANCE

$TABLE ID TIME DV SIZE MDV Y SIGM MU ETA(1) ETA(2) CUMEVT CWRES IPRED NOPRINT ONEHEADER FILE=CD.txt

**NONMEN codes of all cause of death model:**

$PROBLEM EFFECT

$DATA ACD.csv IGNORE=C

$INPUT ID TIME DV SIZE MDV

$SUBROUTINE ADVAN6 TOL=4

$MODEL COMP=(HZLAST)

$PK

SIGM=THETA(1)*EXP(ETA(1))

MU=THETA(2)*EXP(ETA(2))

$DES

DEL= 1E-12

TIM=T+DEL

LNT = LOG(TIM)

X1 =(LNT-MU)/SIGM

PDF= EXP(-1/2*(X1**2))/SQRT(2*3.14159265)

DADT(1)=1/(TIM*SIGM)*PDF/(1-PHI(X1))

$ERROR

CUMEVT=A(1)

EFT=1-EXP(-CUMEVT)

IPRED=EFT

W=SQRT((DV+0.001)*(1-DV+0.001)/SIZE)

Y=EFT+W*ERR(1)

$THETA (0,5) ;SIGM

$THETA (0,6) ;MU

$OMEGA 0,FIXED

$OMEGA 0.09

$SIGMA 1

$ESTIMATION METHOD=1 INTER MAXEVAL=9990 PRINT=10 NOHABORT NOTHETABOUNDTEST NOOMEGABOUNDTEST NOSIGMABOUNDTEST POSTHOC

$COVARIANCE

$TABLE ID TIME DV SIZE MDV Y SIGM MU ETA(1) ETA(2) CUMEVT CWRES IPRED NOPRINT ONEHEADER FILE=ACD.txt

**NONMEN codes of hospitalized for heart failure model:**

$PROBLEM EFFECT

$DATA HHF.csv IGNORE=C

$INPUT ID TIME DV SIZE MDV

$SUBROUTINE ADVAN6 TOL=4

$MODEL COMP=(HZLAST)

$PK

SIGM=THETA(1)+ETA(1)

MU=THETA(2)*EXP(ETA(2))

$DES

DEL= 1E-12

TIM=T+DEL

LNT = LOG(TIM)

X1 =(LNT-MU)/SIGM

PDF= EXP(-1/2*(X1**2))/SQRT(2*3.14159265)

DADT(1)=1/(TIM*SIGM)*PDF/(1-PHI(X1))

$ERROR

CUMEVT=A(1)

EFT=1-EXP(-CUMEVT)

IPRED=EFT

W=SQRT((DV+0.001)*(1-DV+0.001)/SIZE)

Y=EFT+W*ERR(1)

$THETA (0,3) ;SIGM

$THETA (0,7) ;MU

$OMEGA 0,FIXED

$OMEGA 0.09

$SIGMA 1

$ESTIMATION METHOD=1 INTER MAXEVAL=9990 PRINT=10 NOHABORT NOTHETABOUNDTEST NOOMEGABOUNDTEST NOSIGMABOUNDTEST POSTHOC

$COVARIANCE

$TABLE ID TIME DV SIZE MDV Y SIGM MU ETA(1) ETA(2) CUMEVT CWRES IPRED NOPRINT ONEHEADER FILE=HHF.txt

**NONMEN codes of change from baseline in 6MWD model:**

$PROBLEM placebo effect

$DATA 6MWD.csv IGNORE=C

$INPUT ID TIME DV SIZE MDV

$PRED

TVB0=THETA(1)

B0=TVB0*EXP(ETA(1))

EFT=B0*TIME

W=1/SQRT(SIZE/100)

IPRED=EFT

Y=EFT+ERR(1)*W

$THETA (-1,0.2) ;B0

$OMEGA 0.09

$SIGMA 1

$ESTIMATION METHOD=1 INTER MAXEVAL=9990 PRINT=10 POSTHOC

$COVARIANCE

$TABLE ID TIME DV SIZE MDV B0 CWRES IPRED ETA1

NOPRINT ONEHEADER FILE=6MWD.txt
